# Initial Vancomycin Taper for the Prevention of Recurrent *Clostridioides difficile* Infection: A Randomized Clinical Trial

**DOI:** 10.1001/jamanetworkopen.2025.60495

**Published:** 2026-02-27

**Authors:** Emily G. McDonald, Guillaume Butler-Laporte, James M. Brophy, Sarah Elsayed, Charles Frenette, Iman Huseen, Vivian G. Loo, Kristen Moran, Bryan Coburn, Susy S. Hota, Yves Longtin, Ling Y. Kong, Matthew P. Muller, Theodore S. Steiner, Louis Valiquette, Nick Daneman, Peter Daley, Caroline Nott, Derek R. MacFadden, Christopher E. Kandel, Yan Chen, Santiago Perez-Patrigeon, Todd C. Lee

**Affiliations:** 1 Division of General Internal Medicine, Department of Medicine, McGill University, Montréal, Quebec, Canada; 2 Clinical Practice Assessment Unit, Department of Medicine, McGill University, Montréal, Quebec, Canada; 3 Division of Infectious Diseases, Department of Medicine, McGill University, Montréal, Quebec, Canada; 4 Department of Epidemiology, Bioethics, and Occupational Health, McGill University, Montréal, Quebec, Canada; 5 Division of Infectious Diseases, Department of Medicine, University Health Network, Toronto, Ontario, Canada; 6 Division of Infectious Diseases, Department of Medicine, The University of Toronto, Toronto, Ontario, Canada; 7 Division of Infectious Diseases, Department of Medicine, University of British Columbia, Vancouver, Canada; 8 Department of Microbiology and Infectious Diseases, Université de Sherbrooke, Sherbrooke, Quebec, Canada; 9 Division of Infectious Diseases, Department of Medicine, Memorial University, St John’s, Newfoundland, Canada; 10 Division of Infectious Diseases, Department of Medicine, University of Ottawa, Ottawa, Ontario, Canada; 11 The Ottawa Hospital Research Institute, University of Ottawa, Ottawa, Ontario, Canada; 12 Department of Medicine, Michael Garron Hospital, Toronto, Ontario, Canada; 13 Department of Laboratory Medicine and Pathobiology, Temerity Faculty of Medicine, University of Toronto, Toronto, Ontario, Canada; 14 Division of Infectious Diseases, Department of Medicine, Queen’s University, Kingston, Ontario, Canada

## Abstract

**Importance:**

*Clostridioides difficile* infection (CDI) is associated with substantial morbidity and mortality, and recurrent CDI (rCDI) is common.

**Objective:**

To determine whether a 4-week vancomycin pulse and taper regimen would be superior to a standard 2-week vancomycin pulse regimen in terms of recurrence.

**Design, Setting, and Participants:**

This parallel-design, double-blind clinical trial was performed at 12 Canadian hospitals. Adults with a first episode or first recurrence of CDI were eligible to participate. Patients needed to have clinical CDI with laboratory confirmation and to have improved by day 10 of treatment. Recruitment began November 19, 2020, and all follow-up was completed by October 4, 2024.

**Intervention:**

All patients received a 2-week pulse of vancomycin (standardized at 125 mg orally 4 times a day at the time of recruitment) and were then randomized to receive either vancomycin taper (125 mg orally twice a day for 7 days and then 125 mg once a day for 7 days) or an equivalent schedule of placebo capsules.

**Main Outcomes and Measures:**

The primary outcome was the posterior probability of superiority of the vancomycin pulse and taper regimen to prevent rCDI at day 56. A secondary outcome was recurrence at day 38. Binary outcomes were analyzed using a bayesian generalized linear model with minimally informative priors yielding log relative risk (RR) with 95% bayesian credible intervals (CrIs).

**Results:**

The trial was stopped early due to feasibility of recruitment. Among 265 participants (135 in the intervention group and 130 in the control group; median age, 63 [IQR 47-74] years; 138 [52.1%] women), recurrence at day 56 occurred in 20 of 135 patients (14.8%) in the vancomycin pulse and taper group compared with 23 of 130 (17.7%) in the vancomycin pulse group (adjusted RR, 0.84 [95% CrI, 0.48-1.45]; posterior probability of superiority, 73.8%). Recurrence at day 38 occurred in 9 of 135 patients (6.7%) in the vancomycin pulse and taper group compared with 20 of 130 (15.4%) in the vancomycin pulse group (adjusted RR, 0.43 [95% CrI, 0.19-0.89]; posterior probability of superiority, 99.0%). Adverse effects were rare in both groups.

**Conclusions and Relevance:**

In this randomized clinical trial, a 4-week vancomycin pulse and taper regimen had a probability of 73.8% to be superior to a 2-week pulse regimen. This approach may represent a safe and accessible treatment option to delay or prevent early CDI recurrence.

**Trial Registration:**

ClinicalTrials.gov Identifier: NCT04138706

## Key Points

**Question:**

Does a 4-week pulse and taper regimen of vancomycin reduce the risk of recurrent *Clostridioides difficile* infection compared with a standard pulse regimen among patients with a first episode or first recurrence?

**Findings:**

In this randomized clinical trial of 265 participants, the posterior probability of superiority for vancomycin pulse plus taper was 74%. The trial was stopped early due to feasibility of recruitment.

**Meaning:**

The vancomycin pulse and taper regimen could represent a safe and accessible treatment option to delay or prevent early recurrence of *C difficile* infection.

## Teaser

This randomized clinical trial tested whether a 4-week pulse and taper vancomycin regimen was superior to a 2-week pulse regimen for preventing recurrence among patients with a first episode or first recurrence of *Clostridioides difficile* infection.

## Introduction

There are more than 450 000 cases of *Clostridioides difficile* infection (CDI) annually in the US and another 37 000 cases in Canada with an estimated combined annual cost approaching $5 billion.^1,2,3,4^ Even with successful treatment, approximately 20% to 25% of patients develop recurrent CDI (rCDI) within 8 weeks.^5,6^ The morbidity and mortality of recurrences^7^ are estimated to cost $2.8 billion in the US^8^ and $65 million annually in Canada.^4^ Consequently, there is an urgent need for strategies to prevent rCDI.

One treatment option to prevent recurrence is fidaxomicin, having demonstrated a clinically significant reduction in the risk of rCDI compared with 10 days of vancomycin monotherapy (15.4% vs 25.3%) for patients experiencing a first or a second episode of CDI.^9^ In the fidaxomicin randomized clinical trials,^9,20^ a more pronounced difference in relapse was observed within 14 days of stopping vancomycin therapy (7.4% for fidaxomicin vs 19.3% for vancomycin), whereas later, recurrence rates were similar (8.1% for vancomycin vs 6.6% for fidaxomicin).^10^ This is relevant as the duration of vancomycin treatment for CDI arose from limited animal and observational data, potentially derived from a single case report of resolution following 10 days of treatment.^11^ Furthermore, despite the availability of fidaxomicin and newer treatments for CDI during the last 10 to 15 years, a recent international survey found that more than 66% of clinicians still use vancomycin for the initial treatment of CDI.^12^

Longer vancomycin pulse and taper regimens are used in the treatment of multiple recurrences of CDI and are effective for preventing recurrence following initial episodes of *C difficile* in the in vitro gut model^13^ and in retrospective cohort studies (mean treatment duration of 25 days).^14^ Recognizing that fidaxomicin is not universally available and is more expensive than vancomycin even after accounting for lower recurrence,^15^ we sought to determine whether a 4-week pulse and taper vancomycin regimen would be superior for preventing rCDI when compared with a 2-week pulse vancomycin regimen.

## Methods

### Trial Design and Oversight

The Initial Vancomycin Taper for the Prevention of Recurrent Clostridium Difficile Infection (TAPER-V) was a parallel-design double-blind clinical trial. TAPER-V began recruitment November 19, 2020, and all follow-up was completed by October 4, 2024. The protocol was approved by local and/or provincial research ethics boards at all participating sites with the Research Institute of the McGill University Health Centre serving as the board of record. The trial was conducted with a “no objection letter” from Health Canada. The protocol and statistical analysis plan are included in Supplement 1. We followed the Consolidated Standards of Reporting Trials (CONSORT) reporting guideline. All patients or their substitute decision makers provided written informed consent. The Clinical Practice Assessment Unit of McGill University coordinated and conducted the trial and collected the data.

### Patients

Patients who tested positive for *C difficile* at participating centers were screened between days 7 to 10 of treatment to identify those meeting eligibility criteria. Inclusion criteria were being 18 years or older and undergoing treatment for a first episode or first recurrence of CDI. CDI was defined as 3 or more episodes of diarrhea in 24 hours with positive results of one of the following: a positive nucleic acid amplification test (NAAT); toxin A/B enzyme immunoassay; or cell cytotoxicity assay. Patients who had less than 3 bowel movements could be included if they had a positive test result in the setting of ileus, toxic megacolon, or pseudomembranous colitis on colonoscopy results.

Exclusion criteria included failure to achieve clinical cure by the end of day 10 (>3 unformed stools per day for 2 days and/or unresolved toxic megacolon); the use of metronidazole monotherapy for more than 3 days; receipt of fidaxomicin, fecal microbiota transplant, or immunoglobulin products for the current episode; previous or current total or subtotal colectomy; severe allergy or intolerance to oral vancomycin; more than 2 episodes of *C difficile* within 5 years; documented sensorineural hearing loss (other than presbycusis and noise-induced hearing loss); and known or planned pregnancy or breastfeeding. Administrative exclusion criteria are listed in the trial protocol (Supplement 1).

Participants were recruited by local investigators and research personnel after obtaining approval from treating physicians. If a patient met eligibility criteria, trial personnel obtained informed consent and prior to randomization collected baseline data about the patient’s demographic characteristics, comorbid medical conditions, proton pump inhibitor use, and history of concomitant antibiotics. Patients could be recruited in the emergency department, as inpatients, or as outpatients.

### Randomization and Interventions

All patients had the length of their initial vancomycin pulse treatment standardized at 14 total days, which is the longest recommended duration in the 2018 Canadian CDI guidelines.^16^ If a patient was receiving more than 125 mg vancomycin orally 4 times per day, the dose was decreased to 125 mg 4 times daily prior to enrollment. Patients were randomly assigned to start the study taper on day 15: 125-mg vancomycin or placebo capsules were given twice daily for 7 days, then once daily for 7 days. This particular taper was chosen as it represents 2 steps of a commonly used 4-week tapering regimen.^17^ Patients underwent randomization using permuted block (random sizes of 2, 4, 6 or 8), which was stratified by the presence or absence of a prior episode of CDI. The allocation sequence was generated by an independent statistician (T.C.L.) using specialized software.^18^ Blinded coded packages of medications were prepared by a central research pharmacy and stored at satellite pharmacies at each site. After the software revealed the randomization code, the packages were dispensed from the pharmacy to the patient either in person or via express courier. Unblinded pharmacists were otherwise not involved in any trial-related activities. The vancomycin and placebo capsules were purchased from Canadian wholesale suppliers. The treating physicians, trial staff, and patients remained unaware of the randomized assignments. Analysis and data safety monitoring were also conducted while blinded.

### Outcome Measures

The primary outcome was rCDI between days 15 and 56, with day 1 being the first day that vancomycin treatment of the qualifying episode of CDI began. Recurrence used the same definition of CDI as study inclusion, combined with a requirement for the administration of treatment. Recurrence events were adjudicated in duplicate, with the reviewers (E.G.M., S.E., and T.C.L) blinded to the intervention and disagreement resolved by consensus with a third reviewer.

Secondary outcomes at day 28 consisted of adverse effects, including those leading to treatment cessation. At day 38, we compared recurrence rates to allow direct comparison with the fidaxomicin randomized clinical trials.^9,19^ Secondary outcomes within 90 days included: recurrence as defined above; receipt of fidaxomicin, colectomy, or fecal microbiota transplantation; emergency department visits and readmission to hospital; and all-cause mortality. As potential modifiers of the primary outcome, we also recorded receipt of off-study antibiotics and receipt of vancomycin secondary prophylaxis. Quality of life was assessed by the CDI health-related quality of life Cdiff32 questionnaire^20^ at day 56, to be analyzed and reported in a subsequent report.

### Trial Procedures

Patients were evaluated on days 28 and 56 (±3 days) for the primary outcome. Subjects also had weekly check-ins via text message, email, or telephone until day 56, and then every 2 weeks until day 90. Positive responses would trigger a physician visit or in-person trial follow-up as appropriate.

### Data Safety and Monitoring Committee Oversight

The first patient was enrolled November 19, 2020. The data safety and monitoring committee (DSMC) met 2 times after enrollment of the first patient to assess the safety and efficacy of the vancomycin taper as compared with the placebo taper regarding the primary outcome, based on prespecified thresholds in the statistical analysis plan (Supplement 1). On November 22, 2024, the DSMC met and following review of the blinded data recommended stopping the trial although the statistical stopping criteria had not been met. Reasons included: (1) the event rates in both arms were lower than anticipated, reducing the power of the study to demonstrate the estimated effect size, and (2) changes to provincial CDI guidelines had repositioned fidaxomicin as first-line therapy for higher-risk patients in the province of Quebec. As the Quebec sites recruited the most patients, it was believed that recruitment to the full sample size was no longer feasible. The DSMC recognized the research question remained scientifically valid and of interest in regions where fidaxomicin was unavailable or not adopted as first-line therapy.

### Statistical Analysis

The initial design assumed an event rate of 25% in the control group and 15% in the intervention group based on fidaxomicin randomized clinical trials.^9,19^ With 80% power and an overall 2-sided α = .05, the trial would require 496 (rounded to 500) patients to demonstrate superiority. After the recruitment of 50 patients, the analytic plan was modified to use a bayesian framework on April 3, 2023, prior to completion of study enrollment and before data were locked and blinded for analysis. This allowed for earlier stopping while preserving the overall type I error rate and maximal sample size in response to an update to the Infectious Diseases Society of America guidelines for the treatment of CDI, which were changed to recommend fidaxomicin as first-line therapy in place of vancomycin.^21^ As the study results could have substantial clinical and financial^15^ implications, integrating early stopping rules was salient. We used the adaptr package, version 1.2.0 (R Project for Statistical Computing),^22^ to simulate 50 000 two-armed trials under both the null and alternative hypotheses using the funded 500 patient sample size and interim analyses at 125, 250, and 500 patients. The thresholds for superiority (relative risk [RR], <1.00) were 99.0% at the first analysis and 97.5% at subsequent analyses. A futility analysis was included whereby the trial would be stopped if there was less than a 15% probability of at least a 4% absolute risk reduction (number needed to treat, ≤25). These simulations estimated an overall type I error rate of 5.1% with 72.6% power and a median expected sample size of 250 patients. If the intervention showed a posterior probability of efficacy or futility by crossing a boundary, the DSMC could recommend stopping the trial.

We applied a bayesian framework to all analyses and reported the posterior probabilities for superiority of vancomycin to placebo for the primary and secondary outcomes. The posterior probability determines how likely it is that the new treatment is better, given the available data. The characteristics of the patients at baseline are reported as counts and percentages or, for continuous variables, as medians with IQRs. All binary outcomes were analyzed using a bayesian generalized linear model (binomial family, log link), which yields log RR with 95% bayesian credible intervals (CrI). These were exponentiated to give risk ratios. Priors were minimally informative (*N* ~ [0, 100^2^]). Stratification was included in the model,^23^ and we ran 4 chains of 50 000 Markov chain Monte Carlo simulations. Exploratory subgroup analyses were assessed in accordance with the prespecified statistical analysis plan included in Supplement 1. Post hoc analysis using restricted mean survival time was suggested by the independent DSMC statistician and is presented in eTable 1 in Supplement 2 using frequentist methods.^24^ Post hoc analyses adjusting for secondary antibiotic exposure and accounting for the site of enrollment were conducted at the request of peer reviewers and are presented in eAppendices 1 and 2 in Supplement 2. Analyses were conducted in Stata, version 17.0 (StataCorp LLC).

## Results

### Trial Population

**Figure 1.**
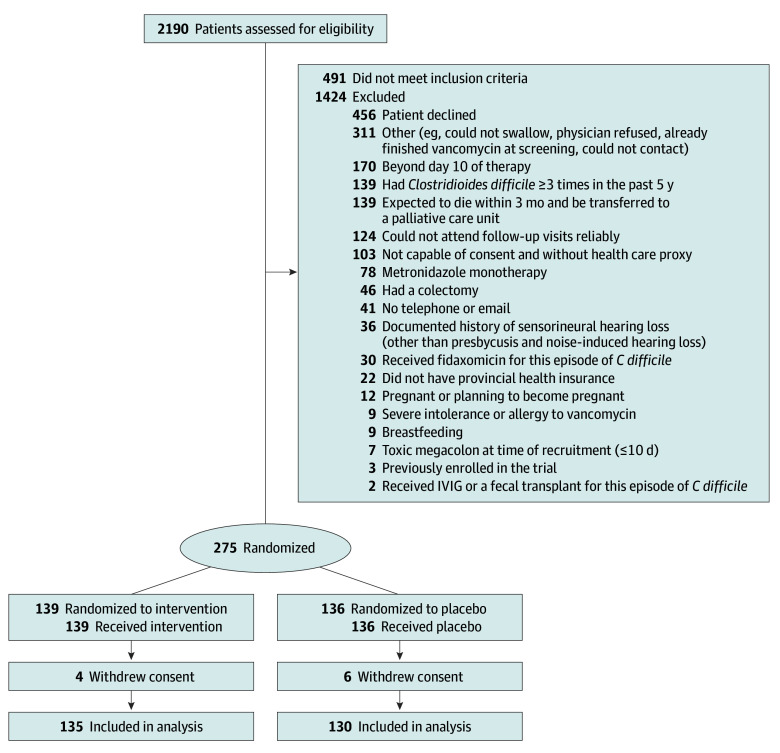
Study Flow Diagram The intervention group was randomized to a vancomycin pulse and taper regimen; the placebo group was randomized to a vancomycin pulse regimen. Patients could be excluded for more than 1 reason. IVIG indicates intravenous immunoglobulin.

A total of 2190 potential participants were screened for inclusion, of whom 275 were recruited and were randomized. Of 275 patients, 139 were randomly assigned to receive vancomycin and 136 to receive placebo (Figure 1). Four and 6 participants, respectively, withdrew from the trial, did not consent to retain their data, leaving 265 patients to be included in the analysis (median age, 63 [IQR, 47-74] years; 138 [52.1%] women and 127 [47.9%] men). Two hundred forty-five included patients (92.5%) were experiencing their first episodes of CDI. Table 1 includes other baseline clinical details.

**Table 1.** Demographic and Clinical Characteristics at Baseline

Characteristic	Patient group, No. (%)^a^		
	Intervention (n = 135)	Placebo (n = 130)	All (n = 265)
Age, median (IQR), y	63 (44-76)	62 (50-76)	63 (47-74)
Sex			
Female	77 (57.0)	61 (46.9)	138 (52.1)
Male	58 (43.0)	69 (53.1)	127 (47.9)
Language			
English	99 (73.3)	90 (69.2)	189 (71.3)
French	36 (26.7)	40 (30.8)	76 (28.7)
Setting of diagnosis			
Outpatient	40 (29.6)	40 (30.8)	80 (30.2)
ED	50 (37.0)	34 (26.2)	84 (31.7)
Hospital	45 (33.3)	56 (43.1)	101 (38.1)
Comorbidities			
Proton pump inhibitor use	62 (45.9)	57 (43.8)	119 (44.9)
Solid organ cancer	22 (16.3)	28 (21.5)	50 (18.9)
Without metastases	14 (10.4)	19 (14.6)	33 (12.5)
With metastases	8 (5.9)	9 (6.9)	17 (6.4)
Hematologic cancer	13 (9.6)	9 (6.9)	22 (8.3)
With transplant	3 (2.2)	2 (1.5)	5 (1.9)
Solid organ transplant	14 (10.4)	18 (13.8)	32 (12.1)
Inflammatory bowel disease	8 (5.9)	21 (16.2)	29 (10.9)
CDI details			
Diagnosis by EIA for toxin	2 (1.5)	4 (3.1)	6 (2.3)
First episode	124 (91.9)	121 (93.1)	245 (92.5)
Concurrent antibiotics at enrollment	23 (17.0)	24 (18.5)	47 (17.7)
Cotreatment			
Nonstudy antibiotics	21 (15.6)	33 (25.4)	54 (20.4)
Open-label vancomycin prophylaxis	16 (11.9)	15 (11.5)	31 (11.7)

Abbreviations: CDI, *Clostridium difficile* infection; ED, emergency department; EIA, enzyme immunoassay.

^a^ The intervention group was randomized to a vancomycin pulse and taper regimen; the placebo group was randomized to a vancomycin pulse regimen.

### Primary Outcome

In the intention-to-treat population, 56-day recurrence occurred in 20 of 135 patients (14.8%) patients in the intervention group, as compared with 23 of 130 (17.7%) in the placebo group (adjusted RR, 0.84 [95% CrI, 0.48-1.45]; posterior probability of superiority, 73.8%) (Table 2).

**Table 2.** Primary and Secondary Outcomes

Characteristic	Patient group, No. (%)^a^		Adjusted relative risk (95% CrI)	Posterior probability of superiority, %
	Intervention group (n = 135)	Placebo group (n = 130)		
Recurrence of CDI				
Day 56 (primary outcome)	20 (14.8)	23 (17.7)	0.84 (0.48-1.45)	73.8
Day 38 (secondary outcome)	9 (6.7)	20 (15.4)	0.43 (0.19-0.89)	99.0
Day 90 (secondary outcome)	23 (17.0)	24 (18.5)	0.92 (0.54-1.56)	62.1
Complications by day 90				
All-cause mortality	4 (3.0)	2 (1.5)	3.91 (0.44-34.50)	9.8
Colectomy	0	0	NA	NA
ED or readmission to hospital	22 (16.3)	26 (20.0)	0.82 (0.48-1.38)	78.3
ED or readmission to hospital for CDI	3 (2.2)	1 (0.8)	2.85 (0.30-27.01)	16.4
Secondary CDI therapies by day 90				
Fecal microbiota transplant	0	0	NA	NA
Receipt of fidaxomicin	1 (0.7)	4 (3.1)	0.19 (0.01-1.47)	94.2

Abbreviations: CDI, *Clostridium difficile* infection; ED, emergency department; NA, not applicable.

^a^ The intervention group was randomized to a vancomycin pulse and taper regimen; the placebo group was randomized to a vancomycin pulse regimen.

### Secondary Outcomes

**Figure 2.**
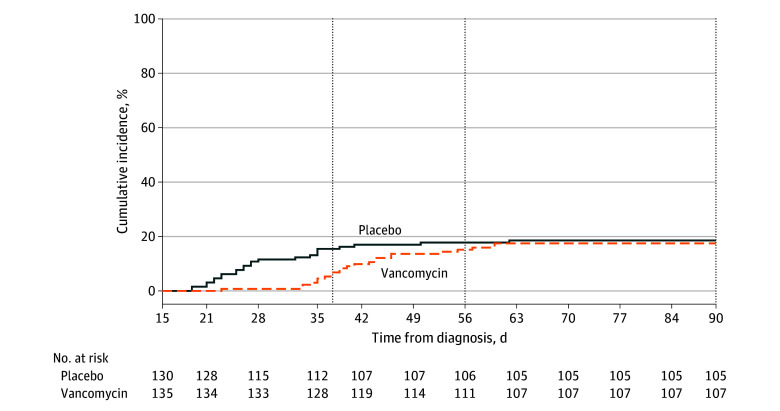
Kaplan-Meier Curve of *Clostridium difficile* Infection Recurrence Over Time The intervention group was randomized to a vancomycin pulse and taper regimen; the placebo group was randomized to a vancomycin pulse regimen.

Most recurrences occurred within the first 2 weeks after stopping vancomycin treatment (Figure 2), with only 1 recurrence (0.7%) during therapy in the vancomycin pulse and taper group. At day 38, corresponding to the follow-up of the fidaxomicin randomized clinical trials,^9,19^ 9 of 135 patients (6.7%) in the vancomycin pulse and taper group had experienced recurrence compared with 20 of 130 (15.4%) in the vancomycin pulse group (adjusted RR, 0.43 [95% CrI, 0.19-0.89]; posterior probability of superiority, 99.0%). Other secondary outcomes are reported in Table 2. The analysis using restricted mean survival time found a modest benefit in favor of the vancomycin pulse and taper regimen of 2.19 (95% CI, 0.51-3.87) days (*P* = .01) (eTable 1 in Supplement 2**)**. Most recurrences occurred as outpatients, and there was a low rate of emergency department visit or hospitalization in both arms, with only 4 *C difficile*–related visits.

### Safety

Adverse effects occurred in 15 of 132 patients (11.4%) in the vancomycin pulse and taper group and 13 of 129 (10.1%) in the vancomycin pulse group (Table 3). These were generally mild, with discontinuation due to adverse effects occurring in 2 (1.5%) and 3 (2.3%) patients, respectively.

**Table 3.** Adverse Effects and Events

Outcome	Patient group, No. (%)^a^	
	Intervention (n = 132)	Placebo (n = 129)
Any adverse effect^b^	15 (11.4)	13 (10.1)
Stopped study drug due to effect	2 (1.5)	3 (2.3)
Required health care contact due to effect	1 (0.8)	1 (0.8)
Gastrointestinal		
Heartburn and/or reflux	1 (0.8)	1 (0.8)
Abdominal discomfort	3 (2.3)	3 (2.3)
Nausea	4 (3.0)	1 (0.8)
Diarrhea	2 (1.5)	3 (2.3)
Mouth sores	1 (0.8)	0
Nongastrointestinal		
Headache	1 (0.8)	1 (0.8)
Light-headedness	0	2 (1.6)
Tingling	0	1 (0.8)
Drowsiness	1 (0.8)	0
Vertigo	0	0
Tinnitus	0	0
Rash	2 (1.5)	2 (1.6)
Pruritus	0	1 (0.8)
Leg swelling	1 (0.8)	0
Upper respiratory tract infection symptoms	1 (0.8)	1 (0.8)

^a^ The intervention group was randomized to a vancomycin pulse and taper regimen; the placebo group was randomized to a vancomycin pulse regimen.

^b^ Adjusted risk ratio, 1.12 (95% CrI, 0.55-2.29); probability of superiority, 38.1%.

### Subgroup Analyses

Exploratory subgroup analyses are included in the eFigure in Supplement 2 and were generally consistent with the overall trial results. The per protocol analyses are presented in eTable 2 in Supplement 2 and were consistent with the intention-to-treat analysis.

## Discussion

This placebo-controlled randomized clinical trial found a 73.8% probability that a 4-week pulse and taper vancomycin regimen reduced the risk of CDI recurrence at day 56 and a 99.0% probability of reduction at day 38. The observed reduction in the probability of superiority from 99.0% at day 38 to 73.4% at day 56 as visualized by the Kaplan-Meier survival curve suggests that some recurrences were delayed as opposed to prevented.

While the initial vancomycin treatment duration for CDI may have been established based on the timing of initial clinical cure,^11^ by 1980 it was obvious that recurrence was a common phenomenon.^25^ It was not until use of fidaxomicin that clinical trials achieved an adequate sample size to adequately study recurrence (as opposed to initial clinical cure).^9^ A possible limitation of the fidaxomicin trials was the shorter follow-up period when compared with the recurrence definition of 56 days of the Society for Healthcare Epidemiology of America and the Infectious Diseases Society of America.^17^ It remains unknown from placebo-controlled trials whether or not fidaxomicin delays or prevents recurrence over a longer time frame. Based on these trials, treatment guidelines^21,26^ have been updated to favor fidaxomicin because of the reduced risk of recurrence when compared with 10 days of vancomycin treatment. Nonetheless, most clinicians worldwide still use vancomycin for the initial treatment of CDI, even in the presence of risk factors for recurrence.^12^ This may be because access to fidaxomicin is not universal and the incremental drug costs remain an important consideration.^15^ The vancomycin pulse and taper regimen could be an option for cases where fidaxomicin is not accessible, and/or where delaying recurrence is important (eg, among more frail, older, patients with more comorbidities). In support of this approach, a recent RCT of a vancomycin pulse and taper regimen vs 10 days of vancomycin for rCDI (first or second episode) presented in abstract form also found a pulse and taper was superior to 10 days of vancomycin for sustained clinical response at day 59.^27^ Importantly, oral vancomycin is considered safe; however antimicrobial resistance has been postulated as a risk of therapy. A propensity-matched cohort study of 82 405 patients treated for CDI with vancomycin or metronidazole did not detect an increased risk of vancomycin-resistant enterococcus in those receiving oral vancomycin.^28^

### Limitations

A major limitation of this trial is that it was stopped early, and the power to demonstrate a benefit at day 56 was consequently reduced. It is possible that the benefits shown at day 38 would have translated into a demonstrable residual benefit at day 56 had we achieved the planned sample size. Another limitation was that both initial and recurrence testing was mainly performed by NAAT in patients with clinical symptoms. Some have advocated for 2-stage testing with enzyme-linked immunosorbent assay to reduce false-positive findings vs NAAT alone^29^; however, in a systematic review including 12 737 patients, the 30-day mortality was not significantly different between those who did and did not have positive toxin findings, and furthermore, the treatment of cases with negative enzyme-linked immunosorbent assay toxin-positive findings were associated with a reduction in mortality.^30^ Therefore, the use of NAAT in patients who meet a clinical case definition in combination with strict submission criteria (eg, laboratory rejection of formed specimens) is still considered an acceptable diagnostic standard.^29,31^ Importantly, the placebo-controlled nature of the trial minimized clinical bias with respect to testing and treatment decisions. Another limitation was the use of an initial 14-day pulse of vancomycin therapy in the control group, which may have biased our findings toward the null. Given the time dependence of recurrence,^10^ it is likely that compared with a 10-day pulse of baseline therapy, we would have demonstrated greater probability of superiority. Finally, use of a longer taper may have further delayed or ultimately prevented recurrences. We opted for a 4-week pulse and taper regimen to limit the impact of a longer regimen on the microbiome.

## Conclusions

For this randomized clinical trial of patients with first episode or first recurrence of CDI, a 4-week vancomycin pulse and taper regimen had a 73.8% probability of being superior to a 14-day pulse therapy for CDI recurrence at day 56, which may have simply been due to delayed recurrences. This approach may represent a safe and accessible treatment option to delay or prevent early CDI recurrence. Further pragmatic randomized evidence will be essential to optimizing therapy for CDI, particularly a head-to-head clinical trial of a vancomycin pulse and taper regimen vs fidaxomicin with longer term follow-up.

## Notes

Supplement 1.
Trial Protocol

Supplement 2. **eTable 1.** Restricted Mean Survival Time Analysis **eTable 2.** Per-Protocol Analyses and Recurrence Outcomes **eFigure.** Exploratory Subgroup Analysis **eAppendix 1.** Post Hoc Analysis: Antibiotic Re-exposure **eAppendix 2.** Post Hoc Analysis: Accounting for Site

Supplement 3.
Nonauthor Collaborators

Supplement 4.
Data Sharing Statement
