# Supplementary material for: Initial Vancomycin Taper for the Prevention of Recurrent Clostridioides difficile Infection: A Randomized Clinical Trial
Source: JAMA Netw Open. 2026 Feb 27;9(2):e2560495. doi: 10.1001/jamanetworkopen.2025.60495 (PMC12949445; doi:10.1001/jamanetworkopen.2025.60495)

Supplemental Appendix for Initial Vancomycin Taper for the Prevention of Recurrent *Clostridioides difficile* Infection: The TAPER-V Randomised Controlled Trial

Table of Contents:

TAPER-V Protocol History of Changes **6**

Taper-V Protocol Version 3.6 July 4, 2024 .... **7**

Section 2: Background Information **9**

2.1 – Name and Description of investigational product(s). 9

2.2 - Summary of findings from nonclinical studies that potentially have clinical significance and from clinical trials that are relevant to the trial. 9

Rationale for Doses and Duration Selected 10

2.3 Summary of the known and potential risks and benefits, if any, to human subjects. 11

Risks 11

Benefits 12

2.4 Description of and justification for the route of administration, dosage, dosage regimen, and treatment period(s). 12

2.5 This trial will be conducted in accordance with the protocol, GCP, and the applicable laws and provincial/national regulatory requirements. 13

2.6 Description of the population to be studied. 13

Inclusion 13

Exclusion 13

Withdrawal Criteria 14

Subject Replacement 14

2.7 References provided at end of document 14

Section 3 Trial Objectives and Purpose **15**

Section 4 Trial Design **16**

4.1 A specific statement of the primary endpoints and the secondary endpoints, if any, to be measured during the trial. 16

Primary outcomes 16

Secondary Outcomes 16

4.2 A description of the type/design of trial to be conducted (e.g., double-blind, placebo-controlled, parallel design) and a schematic diagram of trial design, procedures and stages. 16

4.3 A description of the measures taken to minimize/avoid bias, including: 17

(a) Randomization 17

(b) Blinding 17

4.4 A description of the trial treatment(s) and the dosage and dosage regimen of the investigational product(s). Also include a description of the dosage form, packaging, and labelling of the investigational product(s). 18

4.5 The expected duration of subject participation, and a description of the sequence and duration of all trial periods, including follow-up, if any. 18

TRIAL SCHEDULE: 18

4.6 A description of the "stopping rules" or "discontinuation criteria" for individual subjects, parts of trial and entire trial. 19

Individual subjects 19
Entire Trial 19
4.7 Accountability procedures for the investigational product(s), including the placebo(s) and comparator(s), if any. 20
4.8 Maintenance of trial treatment randomization codes and procedures for breaking codes. 20
4.9 The identification of any data to be recorded directly on the CRFs (i.e., no prior written or electronic record of data), and to be considered to be source data. 20

Section 5 – Selection and Withdrawal of Subjects 21
5.1 Subject inclusion criteria. 21
5.2 Subject exclusion criteria. 21
5.3 Subject withdrawal criteria (i.e., terminating investigational product treatment/trial treatment) and procedures specifying: 21
When and how to withdraw subjects from the trial/ investigational product treatment. 21
The type and timing of the data to be collected for withdrawn subjects. 21
Whether and how subjects are to be replaced. 21
The follow-up for subjects withdrawn from investigational product treatment/trial treatment. 21

Section 6 – Treatment of Subjects: 22
6.1 The treatment(s) to be administered, including the name(s) of all the product(s), the dose(s), the dosing schedule(s), the route/mode(s) of administration, and the treatment period(s), including the follow-up period(s) for subjects for each investigational product treatment/trial treatment group/arm of the trial. 22
6.2 Medication(s)/treatment(s) permitted (including rescue medication) and not permitted before and/or during the trial. 22
6.3 Procedures for monitoring subject compliance. 22

Section 7 – Assessment of Efficacy 23
7.1 Specification of the efficacy parameters. 23
7.2 Methods and timing for assessing, recording, and analysing of efficacy parameters. 23
C. difficile recurrence (Day 56 – primary outcome; Day 90 – secondary outcome): 23
All cause mortality (90 days): 23

Section 8 – Assessment of Safety 25
8.1 Specification of safety parameters 25
8.2 The methods and timing for assessing, recording, and analysing safety parameters. 25
Discontinuation of study drug (day 28): 25
Other adverse events: 25
Reporting Timeline for SAE/Safety/SUSAR Events: 26
8.3 Procedures for eliciting reports of and for recording and reporting adverse event and intercurrent illnesses. 26
8.4 The type and duration of the follow-up of subjects after adverse events. 26

Section 9 – Statistics 27
9.1 A description of the statistical methods to be employed, including timing of any planned interim analysis. 27
Primary Outcome: 27
Secondary Outcomes: 27
Sensitivity Analyses: 27
9.2 The number of subjects planned to be enrolled. 28
Recruitment rates and loss to follow-up 28

Compliance ... 29
9.3 The level of significance to be used ... 29
9.4 Criteria for the termination of the trial. ... 29
9.5 Procedure for accounting for missing, unused, and spurious data. ... 29
9.6 Procedures for reporting any deviation(s) from the original statistical plan (any deviation(s) from the original statistical plan should be described and justified in protocol and/or in the final report, as appropriate). ... 30
9.7 The selection of subjects to be included in the analyses (e.g., all randomized subjects, all dosed subjects, all eligible subjects, evaluable subjects). ... 30
Intention to treat: ... 30
Per protocol: ... 30

Section 10 – Direct Access to Source Data/Documents ... 30

Section 11 – Quality Control and Quality Assurance ... 31

Section 12 – Ethics ... 31

Section 13 – Data Handling and Record Keeping ... 31

Section 14 – Financing and Insurance ... 32

Section 15 – Publication Policy ... 32

REFERENCES ... 32

TAPER-V Statistical Analysis Plan ... 34

Section 1: Administrative Information ... 34
Table 1. Study information ... 34
1.1 Revision Control ... 35
1.2 Roles and responsibilities ... 35
1.3 Contributions ... 36
1.4 Abbreviations and Definitions ... 36

Section 2: Introduction ... 37
2.1 Background and Rationale ... 37
2.2 Objectives ... 37
2.3 Study Outcome Measures ... 37
2.3.1 Primary Outcome Measures ... 37
2.3.2 Secondary Outcome Measures ... 38
Version 1.2 ... 39

Section 3: Study Methods ... 39
3.1 Trial Design ... 39
3.2 Randomization ... 40
3.2.1 Randomization Procedures ... 40
3.2.2 Masking Procedures ... 40
3.3 Sample Size ... 41
Version 1 ... 41
Version 1.1 ... 41
3.4 Statistical Interim analyses and stopping guidance (if applicable) ... 41
Version 1 ... 41
Version 1.1 ... 42

3.5 Timing of final analysis 42
3.6 Timing of outcome assessment 42

Section 4: Statistical Principles 43
4.1 Confidence Intervals and P-values 43
Version 1 43
Version 1.1 43
4.2 Adherence and protocol deviations 43
4.3 Approach to missing data 43
4.4 Analysis populations 43
Intention to Treat 43
Per Protocol 44
Version 1.2 44
Safety Analysis Population 44

Section 5: Trial Population 44
5.1 Eligibility 44
5.2 Withdrawal/Follow-up 44
5.3 Baseline Patient Characteristics 44

Section 6: Analysis 45
6.1 Statistical analysis of primary and secondary outcomes 45
Primary outcome 45
Version 1 45
Version 1.1 45
Secondary outcomes 45
Version 1 45
Version 1.1 45
6.2 Analysis Methods 46
Sensitivity analyses 46
Version 1 46
Version 1.1 46
Subgroups (presented as forest plots of relative risk for primary outcome) 46
6.3 Missing Data 47
6.4 Harms 47
Side Effects: 47
Version 1.0 47
Version 1.1 47
6.5 Future manuscripts 48

Section 7: References 48

Supplemental Figures and Tables 49

Supplementary Table 1. Participating Sites 49

Supplementary Table 2. Collaborators – included as the “TAPER-V Team” 49

Supplementary Table 3. Data Safety Monitoring Committee 50

Supplementary Table 4. Restricted Mean Survival Time Analysis 51

Supplementary Table 5. Per Protocol Analyses and Recurrence Outcomes 52

Supplementary Figure 1. Time to Recurrence 53
Supplementary Figure 2. Exploratory Subgroup Analysis..54

TAPER-V Protocol History of Changes

Protocol version	Summary of Changes
v1<br>22-Aug-2019	n/a
v1<br>25-Nov-2019	Protocol restructured according to ICH E6/R2 format for submission to Health Canada.
v2<br>16-Jan-2020	Protocol revised according to Health Canada information request
v3<br>28-Aug-2020	Change of study medication from brand name Searchlight Pharma VANCOCIN & custom-made identical placebo to generic JAMP vancomycin and wholesale placebo.
v3.1<br>22-Feb-2021	Changes due to COVID-19 limitations:<br>- Day 28 & 56 in-person visits changed to allow for remote visit (phone or video consultation)<br>- Verbal consent allowed when signed consent not possible<br>Change to Appendix 1: removal of co-investigators table. To be maintained separately.
v3.2<br>07-Apr-2022	- Clarified exclusion criterion is 2 episodes of *C. difficile* in the last 5 years (not lifetime)<br>- Clarified the drug schedule so it is consistent throughout the protocol (Day 1-14: Initial treatment with vancomycin, Day 15-28: study drug (placebo or additional vancomycin)<br>- Changed record retention period from 25 to 15 years as per latest Health Canada guidelines<br>- Corrected typographical errors
v3.3<br>22-June-2022	Added the following specification to the metronidazole monotherapy exclusion criterion:<br>"Participants may be eligible if they are initially treated with metronidazole but switch to oral vancomycin within 3 days (i.e. maximum 3 days of metronidazole monotherapy)." This change was made to accommodate sites where metronidazole may be started empirically before switching to vancomycin when *C. difficile* test result is confirmed.
v3.4<br>03-Apr-2023	Changes pertain to the statistical analysis of the trial, mainly that the analysis will now be conducted using a Bayesian approach and align with the separate statistical analysis plan
v3.5<br>22-Aug-2023	Removed stool sample collection/storage
v3.6<br>04-Jul-2024	Added clarifications to the ITT and per-protocol population definitions to align with the SAP

Taper-V Protocol Version 3.6 July 4, 2024

Initial vancomycin taper for the prevention of recurrent *Clostridium difficile* infection (TAPER-V)

MP-37-2020-5986

Health Canada Submission Protocol – Version 3.6 July 4, 2024

Funding:

Canadian Institutes of Health Research

Sponsor:
Research Institute of the McGill University Health Centre
Nominated Principal Investigator

Todd C. Lee MD MPH
Associate Professor of Medicine, McGill University
1001 Decarie Blvd. E5-1820
Montreal, QC H4A 3J1
514-934-1934x53333

Co-Principal Investigator

Emily G. McDonald MD MSc
Assistant Professor of Medicine, McGill University
Centre for Outcomes Research and Evaluation (CORE)
Office 3E.03, 5252 De Maisonneuve Blvd
Montreal, QC H4A3S5
514-934-1934x53333

Clinical Trials Registration NCT04138706

Statement of Compliance

This protocol will receive independent institutional review board (IRB) permission in each participating site. For the purposes of regulatory compliance, the sponsor institution is the Research Institute of the McGill University Health Center for the operations in Canada.

Signature Page

The signature below constitutes the approval of this protocol and provides the necessary assurances that this trial will be conducted according to all stipulations of the protocol, including all statements regarding confidentiality, and according to local legal and regulatory requirements and applicable Canadian law and ICH guidelines.

Principal Investigator:

Signed: [redacted] Date: 04-JUL-2024

Todd C. Lee
Associate Professor of Medicine, Research Institute of the McGill University Health Center

Section 2: Background Information

2.1 – Name and Description of investigational product(s).

This is a pragmatic, real-world, investigator-initiated placebo-controlled randomized controlled trial where all patients will initially receive standard oral vancomycin dosing (according to the most recent Canadian guidelines[1]) followed by either a two week low dose tapering continuation phase (intervention) or matched placebo (control).

Oral vancomycin is licenced in Canada for the treatment of *C. difficile* and is the preferred treatment in the Canadian guidelines. The differences between this study and the product monograph involve:

1) All patients will receive 14 days of oral vancomycin initially (which is the maximum length suggested in the Canadian guidelines and is the current standard of care at McGill). The monograph states a "usual duration" of 7 to 10 days.
2) The use of a tapering dose of 125mg orally twice daily for 1 week and once daily for 1 week vs. placebo.

The rationale for #1 and #2 will be presented in section 2.2.

The placebo and oral vancomycin to be used for the taper phase will be commercially purchased through existing supply chains. This will involve the JAMP-Vancomycin 125mg capsule from JAMP Pharma Corporation **(DIN 02407744)**.

The placebo will be a B6, B12 and folic acid capsule available on the Canadian market (**NPN 80025487**) from Webber Naturals Pharmaceuticals.

Neither JAMP Pharma nor WN Pharmaceuticals have had any role in the development, design or funding of this project and will have no role in the conduct, analysis and publication.

2.2 - Summary of findings from nonclinical studies that potentially have clinical significance and from clinical trials that are relevant to the trial.

Clostridium difficile infection (CDI) affects nearly 450,000 patients in the United States and 37,000 patients in Canada annually at an estimated annual cost approaching 5 billion dollars.[2-5] It is the most common nosocomial infection, characterized by diarrhea that ranges from mild to fatal.[6,7] Even with successful treatment, approximately 25% of patients develop recurrent CDI (rCDI) within 8 weeks.[8,9] Such recurrences are estimated to cost $2.8 billion in the United

States[10] and $65 million annually in Canada[4] and are associated with morbidity and mortality.[11] Consequently, there is an urgent clinical demand for strategies to prevent rCDI.

The recommended first line therapy for an initial episode of CDI is 10-14 days of oral vancomycin.[1] Although response rates for the treatment of a first episode of CDI now approach 90%,[12-14] approximately 25% of patients who have a complete response will develop rCDI within 8 weeks [8,9,14-16] and our ability to predict recurrence is evolving, but remains very limited.[17] Unfortunately, the incidence of rCDI is increasing at a faster rate than primary CDI[9,18] yet research into rCDI prevention remains underdeveloped and strategies for prevention therefore represent a major knowledge gap. Our study will contribute substantively to our knowledge around initial treatment options and will be of international importance given the costs of recurrence paired with vancomycin's widescale availability as a generic medication with relatively low cost.

Rationale for Doses and Duration Selected

We chose to standardize the initial treatment duration at 14 days (the guidelines have a range 10-14 days) because we did not want to unfairly favor a 2-week tapering regimen by comparing to a regimen which may be too short. The duration of 10 days was itself not scientifically determined through controlled trials; rather, it arose organically from observational data and was initially based on a single case report reporting cure by 10 days[19].

Current vancomycin treatment regimens may leave a residual burden of *C. difficile* which contributes to the risk of recurrence.[20] Extended courses with vancomycin tapering are recommended by Canadian and American[15] guidelines for patients with multiple recurrences (weak recommendation, low quality of evidence). Importantly, tapering is also presented as an option for earlier disease forms in the American guidelines without a supporting citation (weak recommendation, low quality of evidence).[15]

We hypothesize that including a brief vancomycin taper to the initial therapy will be more effective in preventing recurrence than the current standard. This hypothesis has both experimental and clinical plausibility: for an initial episode, extended regimens of vancomycin are effective in preventing recurrence in both the *in vitro* gut model[21] and in retrospective cohort studies (after a mean duration of 25 total days).[20] Our proposal to evaluate the extension of initial treatment from 14 to 28 days with a tapering dose of vancomycin represents a practical clinical trial that capitalizes on oral vancomycin’s safety profile, worldwide availability, and relatively low cost. It will provide much needed support for future guidelines such that future versions can increasingly be based on quality evidence.

Importantly, when comparing vancomycin to fidaxomicin in the trial which led to the licensure of the later, there was a difference in the rate of recurrence in favor of fidaxomicin (15.4% vs. 25.3% at day 36-40)[14]. However, most of the vancomycin failures occurred between Days 11-24 (19.3% vancomycin vs. 7.4% fidaxomicin, $p<0.001$) which suggests that longer initial vancomycin therapy could reduce the risk of recurrence.[22] Importantly, after day 24 the

recurrence rate was similar between the two drugs (8.1% vancomycin vs. 6.6% fidaxomicin, p=0.4).[22] Further, as recurrence was evaluated between days 36-40 in the fidaxomicin trial, it is also uncertain whether the differences would have persisted to 56 days (the most widely accepted definition for recurrence).[15] A delay of the initial recurrence could still be important as it could allow sick patients to recover between episodes but the goal should still remain prevention. Regardless, the cost of fidaxomicin ($220/day) remains prohibitive such that it is not covered on many provincial formularies where generic vancomycin might cost as low as $12/day.

This particular taper was chosen as it represents two steps of a commonly used 4-week vancomycin taper regimen.[23] The study team believed that this dose and duration covers the period of highest risk (as described above) and represents a compromise between the potential efficacy of a longer regimen, antimicrobial stewardship, theoretical "microbiome preservation", and patient compliance.

2.3 Summary of the known and potential risks and benefits, if any, to human subjects.

Risks

Oral vancomycin has a well-established safety profile having been in use for decades in Canada. However, there are important side effects to be aware of. In this study, it is important to recognize that all patients who will be enrolled in this trial would have already been receiving oral vancomycin as the standard of care prior to recruitment for approximately 1 week to ten days. During this period (prior to enrollment) they will have received oral vancomycin at a higher frequency and potentially initially also at higher doses than those being proposed for the taper phase because initial treatment is up to the treating doctors. We believe that most patients will be therefore enrolled after enough drug exposure to vancomycin that efficacy, tolerability and side effects will have already likely have been demonstrated.

We will not be enrolling patients who have firm contra-indications to oral vancomycin since these patients will already be receiving and tolerating oral vancomycin at recruitment.

The vancomycin product monograph lists several known adverse reactions which will be specifically discussed herein:

Nephrotoxicity

Nephrotoxicity has been described in case reports. The causality of renal failure is difficult to determine as most patients will have multiple risk factors for renal insufficiency including acute illness and concomitant administration of other potential causes for renal insufficiency.

Notwithstanding, the total daily doses we have chosen for the taper (250mg total for 1 week then 125mg total for 1 week) are much lower than those expected to pose such a risk. In our informed consent, we do counsel patients for signs and symptoms of renal problems such that they can be brought to medical attention.

Ototoxicity:

This is a case-reportable adverse event which we believe is also unlikely to occur during the taper phase, rather it is more likely to be detected in the early vancomycin exposure phase when they are receiving higher doses and not a yet part of our study. Nonetheless, we counsel patients for signs and symptoms of ototoxicity in our informed consent process such that they can be brought to immediate attention.

Hematopoietic:

Drug-induced neutropenia has been described but almost always after 25g of exposure. In our study, the placebo group will have a total exposure to vancomycin of approximately 7-10g (if they receive larger doses than 125mg in first few days of therapy pre-enrollment). The additional vancomycin exposure in the treatment group will be 2.6g. Therefore, all patients should be well below 25 total grams and this side effect will not be seen.

Drug Reaction with Eosinophilia and Systemic Symptoms Syndrome (DRESS) and Toxic Epidermal Necrolysis (TEN):

This is another rare, idiosyncratic effect which presumably would manifest prior to trial enrollment but may not. We specifically counsel patients for signs and symptoms of rash during our informed consent process.

Miscellaneous:

We believe that these side effects are all more likely to occur pre-enrollment and that the highest risk patients will be excluded prior to enrollment because they will not be receiving oral vancomycin anymore. Our informed consent document discusses many of these severe side effects so that they contact us.

Benefits

We believe that the additional vancomycin given for the treatment of CDI will be associated with a reduced risk of recurrence when compared to placebo.

2.4 Description of and justification for the route of administration, dosage, dosage regimen, and treatment period(s).

Please see section 2.2

2.5 This trial will be conducted in accordance with the protocol, GCP, and the applicable laws and provincial/national regulatory requirements.

2.6 Description of the population to be studied.

The goal of this study will be to have the widest applicability to the care of CDI patients in Quebec, Canada and worldwide. Hence, we will be attempting to include as wide a range of adult patients as possible with minimal exclusion criteria.

This is a multi-centre study involving institutions in British Columbia, Ontario, Quebec and Newfoundland. The study population will be drawn from patients cared for as inpatients or outpatients at the participating hospitals who test positive for *Clostridium difficile* and who tolerate and have an acceptable clinical response to at least 7-10 days of a planned two-week standard of care course of oral vancomycin therapy for CDI or first recurrence of CDI.

The trial will involve only adult patients between the ages of 18 to 100 inclusively.

Inclusion

Essentially, all consecutive adult patients (inpatients and outpatients) who have a treated first episode or first recurrence of CDI (as in the fidaxomicin trial[14]) will be considered.

For enrollment CDI will be defined by a positive PCR for toxin gene and/or detection of toxin by EIA or CCA along with three or more episodes of diarrhea in the 24 hours prior to testing.[14] Patients with a positive test with less than three bowel movements may be included if they initially presented with ileus or if they had pseudomembranous colitis visualized on colonoscopy.

Patients will have achieved clinical cure defined as the resolution of diarrhea (i.e., three or fewer unformed stools for 2 consecutive days)[14] by the date of enrollment (allowing for resolution up to day 10 after diagnosis) and will be tolerating oral vancomycin.

Exclusion

***Clinical*:**

1) Toxic megacolon
2) For the current episode of CDI: use of metronidazole monotherapy*, fidaxomicin, fecal microbiota transplant or intravenous immunoglobulins
   *Participants may be eligible if they are initially treated with metronidazole but switch to oral vancomycin within 3 days (i.e. maximum 3 days of metronidazole monotherapy).
3) Previous or current colectomy
4) Severe allergy/intolerance to oral vancomycin
5) Patient is expected to die within 3 months from another disease or is expected to be admitted to a palliative care unit
6) Failure to achieve clinical cure (as above) by day 10
7) More than 2 episodes of *C. difficile* in the last 5 years

8) Documented history of sensorineural hearing loss (other than presbycusis and noise induced hearing loss). The following patients with documented previous subtypes of sensorineural hearing loss will be excluded from the trial: Menière’s disease, multiple sclerosis affecting auditory nerves, otic syphilis, viral cochleitis, autoimmune disorders, previous drug induced hearing loss, and otherwise unexplained sudden sensorineural hearing loss (SSNHL)
9) Known pregnancy or planning to become pregnant during the study period
10) Women who are breast feeding

Administrative:

1) Expected transfer to a palliative care unit or non-study hospital;
2) No provincial health insurance
3) Previously enrolled
4) No reliable means of outpatient contact
5) Incompetent without healthcare proxy
6) Patient stated inability to come to follow up appointments.

Withdrawal Criteria

The main reason for discontinuation would be allergy or adverse reactions to the study drug. Equally the patient can withdraw at any time. It is possible the trial could be stopped early after data safety and monitoring committee review.

Subject Replacement

Subjects who withdraw for the study or who are lost will be replaced to achieve the necessary sample size.

2.7 References provided at end of document

Section 3 Trial Objectives and Purpose

As discussed in section 2.2, the goal of this study is to determine if a short course taper, when added to the guideline suggested therapy for CDI, is associated with a reduced risk of CDI recurrence. This is an important goal given the burden of rCDI in Canada and worldwide. While fidaxomicin seems to achieve these ends (based on the single RCT), the duration studied in that trial was shorter than the conventional definition and the costs of the drug are financially unsustainable for most health care systems. We hope that the proposed low-cost strategy safely prevents CDI, and, in the case that it merely delays CDI, that those delays are clinically important.

Section 4 Trial Design

4.1 A specific statement of the primary endpoints and the secondary endpoints, if any, to be measured during the trial.

Primary outcomes

C. difficile recurrence within 56 days of initial event.

Secondary Outcomes

- Late recurrence out to 90 days
- Recurrence at day 38 (fidaxomicin outcome)
- Use of fidaxomicin, colectomy or fecal microbiota transplantation within 90 days
- All-cause death within 90 days
- *C. difficile* associated quality of life at day 56
- Emergency Room visit within 90 days
- Re-admission to hospital within 90 days
- Discontinuation of study drug at 28 days
- Receipt of non-study antibiotics within 90 days
- Use of vancomycin secondary prophylaxis within 90 days
- Economic analysis
- Safety & tolerability of the vancomycin extension/taper treatment period (i.e. days 15 -28 inclusive)

4.2 A description of the type/design of trial to be conducted (e.g., double-blind, placebo-controlled, parallel design) and a schematic diagram of trial design, procedures and stages.

This is a placebo-controlled randomized controlled trial comparing tapering vancomycin to placebo for the prevention of *C. difficile* recurrence. Oral vancomycin is approved for the treatment of *C. difficile*. We will also be using dosing which differs from the product monograph to determine if the modified dose improves the chances of cure at 56 days (where cure implies lack of recurrence).

Figure 1 Schematic of Trial

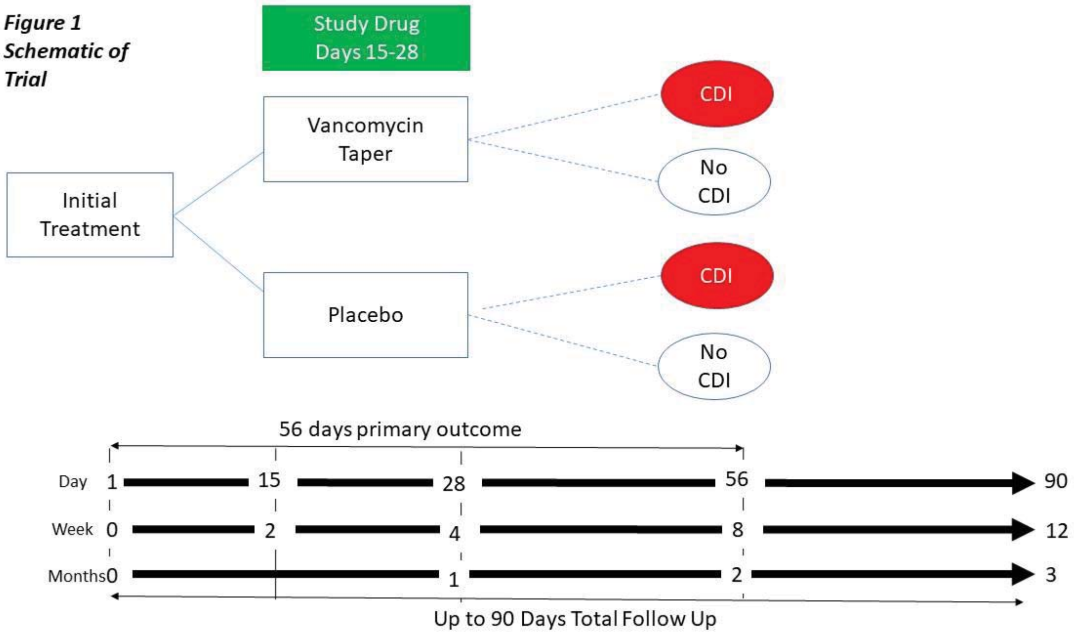

The initial treatment will be counted as 14 calendar days. Day 1 is the first day that the patient receives at least one dose of vancomycin even if they do not receive all 4 doses on that day (ex. due to treatment started in the evening). The last day of initial treatment will be on Day 14, such that the study drug (placebo or additional vancomycin) will be started on Day 15.

4.3 A description of the measures taken to minimize/avoid bias, including:

(a) Randomization

For patients who have enrolled in the study, randomization will occur centrally at McGill University via an existing internet application and will be performed by permuted block with randomized block sizes. This randomization will be stratified for first episode or first recurrence at study entry to ensure these factors are properly balanced.

(b) Blinding

This trial will be placebo controlled using a low dose B6, B12 & folic acid vitamin with an existing NPN (80025487). The supply of vancomycin and placebo capsules will be distributed centrally from the McGill University Health Centre's research pharmacy to each centre's receiving pharmacy. From there they will be dispensed to subjects in sealed packages which are prelabeled according to GCP.

4.4 A description of the trial treatment(s) and the dosage and dosage regimen of the investigational product(s). Also include a description of the dosage form, packaging, and labelling of the investigational product(s).

Please see sections 2.1 and 2.2.

The study drug will be commercially sourced vancomycin from JAMP Pharma Corporation. The placebo will be a low dose vitamin capsule from WN Pharmaceuticals Ltd. as described above.

4.5 The expected duration of subject participation, and a description of the sequence and duration of all trial periods, including follow-up, if any.

Patients will be enrolled between days 7-10 of their initial CDI. They will then be followed with 2 in-person or remote (phone or video consultations) visits at days 28 and 56. They will receive weekly surveys about their health status until Day 56 and then bi-weekly out to day 90.

In the event of a suspected adverse event or CDI recurrence, patients will ideally be seen in person as soon as possible.

TRIAL SCHEDULE:

**Timeline**	**Activity/Treatment**	**Measurements**
Day 1	Patient diagnosed with *C. difficile* and eligible to start or on standard of care oral vancomycin treatment	Determine eligibility and obtain permission for approach
Day 7-10 (Patient's *C. difficile* has improved and meets eligibility)	Consent obtained*; randomization; distribution of study drug for day 15 start	Collection of demographics
Day 15-28	Receipt of study therapy	
Day 28	In-person or remote visit**	
Day 56	In-person or remote visit**	Primary outcome determined, quality of life questionnaire
Day 90	Study ends for the patient	Secondary outcomes can be determined
Weekly until Day 56	Brief questionnaire	By email/text/phone
Biweekly after Day 56	Brief questionnaire	By email/test/phone
Ad hoc	If patient has symptoms of recurrence of *C. difficile*	Review by ID physician in clinic if possible, otherwise usual doctors or emergency room.

Ad hoc	If patient starts antibiotics for another reason	By telephone or in person they will discuss with the study doctor and off-study off-label use of vancomycin secondary prophylaxis will be recorded

* In certain cases where it may not be possible to obtain signed consent (ex. *C. difficile* patients who are also COVID-19 positive or elderly outpatients who are unable to email the signed consent form back), verbal consent may be obtained in the presence of a witness.

** Due to the COVID-19 pandemic, many hospital clinics are now operating on a remote basis by providing phone and video consultations. When medically necessary (ex. In case of recurrence), patients will be seen in-person by a physician.

4.6 A description of the "stopping rules" or "discontinuation criteria" for individual subjects, parts of trial and entire trial.

Individual subjects

If a patient has the primary outcome, they will be treated as medically appropriate and no longer receive study drug. They will still complete the day 56 quality of life questionnaire and have their vital status determined at day 90.

If a patient has a severe adverse event believed to be related to the study drug after urgent assessment by the site investigator or another doctor the patient will immediately stop the study drug. The adverse event will be recorded and reported as required by policy and law. The patient will still complete the remainder of their follow up to determine the other endpoints.

If a patient decides to withdraw, we will ask them to keep the study data accrued to date for the purposes of scientific integrity. We will also ask them if they would consider completing the 90 day follow up electronically.

Entire Trial

We will adopt a Bayesian approach to analysis which maintains a trial-wide type 1 error of approximately 5%. Interim analysis will be performed at 25 and 50% completion and presented to a Data Safety Monitoring Board (blinded). We will use pre-specified rules for stopping the study for efficacy and futility as described later.

4.7 Accountability procedures for the investigational product(s), including the placebo(s) and comparator(s), if any.

All study drug and placebo will be purchased from licenced Canadian commercial sources and involve a drug and natural health product approved in Canada. The study drug is approved for the treatment of CDI in Canada. These will be directly received at McGill University Health Centre and shipped to study sites from there using approved methods of shipping pharmaceutical products.

4.8 Maintenance of trial treatment randomization codes and procedures for breaking codes.

A database of Study ID and treatment assignment are kept centrally and secured electronically. The study will remain blinded until after analysis is complete. However, if there is a specific request for unblinding due to concern for a serious adverse event, particularly one which is unexpected, we have the means of providing emergency unblinding without compromising the study. In this case, the site investigator would request unblinding by study ID and that patient's treatment assignment will be communicated to them in writing.

4.9 The identification of any data to be recorded directly on the CRFs (i.e., no prior written or electronic record of data), and to be considered to be source data.

All required data will be abstracted from the original clinical documents and entered onto our electronic CRFs. We will not keep a copy of the patient's clinical chart separate from the hospital original – and the hospital original chart will be considered the medicolegal copy.

Section 5 – Selection and Withdrawal of Subjects

5.1 Subject inclusion criteria.

See 2.6

5.2 Subject exclusion criteria.

See 2.6

5.3 Subject withdrawal criteria (i.e., terminating investigational product treatment/trial treatment) and procedures specifying:

When and how to withdraw subjects from the trial/ investigational product treatment.

See also section 4.6. A patient who has the primary outcome (recurrence of CDI during the 2-week tapering vancomycin (or placebo) period) or who is suspected of having a treatment associated adverse event requiring discontinuation will be stopped. Ideally, these patients will agree to continue providing outcomes data to protect the integrity of the study.

Subjects can withdraw their consent at any time. However, initial consent asks that if they withdraw, we can keep their study outcomes to date for the purposes of study integrity.

The type and timing of the data to be collected for withdrawn subjects.

We intend to keep basic demographics (as used for Table 1) and outcomes data up to the date of withdrawal.

Whether and how subjects are to be replaced.

If patients withdraw before day 56 without having a primary event they may be replaced.

The follow-up for subjects withdrawn from investigational product treatment/trial treatment.

Clinical follow up with their usual doctors. Study follow-up with the text/email surveys.

Section 6 – Treatment of Subjects:

6.1 The treatment(s) to be administered, including the name(s) of all the product(s), the dose(s), the dosing schedule(s), the route/mode(s) of administration, and the treatment period(s), including the follow-up period(s) for subjects for each investigational product treatment/trial treatment group/arm of the trial.

See section 2.2 for the treatments administered, doses, schedule and route. Follow up will be up to day 56 (primary outcome) and day 90 (secondary outcome).

See also section 4.5 for the trial schedule.

6.2 Medication(s)/treatment(s) permitted (including rescue medication) and not permitted before and/or during the trial.

Cross-over is not allowed. However, if the patient has developed the primary outcome, they may stop the study drug (if taking it) and receive vancomycin again for *C. difficile* if clinically appropriate.

Exposure to antibiotics for other infections and receipt of vancomycin secondary prophylaxis in that context (off label and off study) will be discouraged; however, we cannot stop outside doctors from practicing medicine within their purview. We will ask patients to contact us if this is happening so that the appropriate data can be recorded to account for these effects outside of the study. If vancomycin secondary prophylaxis has occurred prior to day 28, the patient will stop the study drug to avoid excess vancomycin exposure, and this will be recorded.

6.3 Procedures for monitoring subject compliance.

At the day 28 visit we will inquire about adherence and the quantity of pills remaining.

Section 7 – Assessment of Efficacy

7.1 Specification of the efficacy parameters.

1) *C. difficile* recurrence
2) All cause mortality

7.2 Methods and timing for assessing, recording, and analysing of efficacy parameters.

C. difficile recurrence (Day 56 – primary outcome; Day 90 – secondary outcome):

Patients reporting diarrhea will be brought in for an in-person interview or booked for a telephone/video outpatient visit and investigated as appropriate clinically without unblinding. Additionally, patients and/or their proxy will be instructed to contact the study team if they believe they are having a recurrence between contacts. Patients will be able to come be assessed for potential relapse by infectious diseases physicians at each site (who may or may not be a part of the study) or could see their usual doctors. Recurrence will be assessed by clinical record review (chart, laboratory, pharmacy records) and any direct patient interview.

CDI recurrence will be defined by three or more diarrheal stools/24-hour period coupled with a positive PCR for toxin gene or and/or detection of toxin by EIA or CCA and administration of treatment. This is similar to the definition used in the NEJM Fidaxomicin trial.[14] However, to avoid missing severe recurrences: for cases of ileus, toxic megacolon, or pseudomembranous colitis on colonoscopy the test result will be used in the absence of three or more stools.

All cause mortality (90 days):

Patients who arrive at their day 28 and 56 in-person or remote follow-ups are alive at those times and patients who respond to their weekly/bi-weekly text/email/phone surveys are, alive at the time of that response. If a patient misses their day 28 or day 56 in-person or remote visit, we will initially review their hospital file to see if they have died. If this does not show death, we will contact the patient (or their proxy) by telephone to determine why they missed the follow up and reschedule it as required. If we cannot reach the patient or their proxy, and the patient has not been replying to the email/text/phone surveys we will search the obituaries.

After day 56 we will use the survey responses as proof of life. If there is no response to the day 90 survey by day 95, we will first check the hospital file to see if they have died. Then, if vital status is not clear, we will search the obituaries. Then if vital status is still not clear, we will attempt to reach the patient (or their proxy) by telephone. If we cannot still reach the patient, we will send a registered letter to their address. If we still cannot reach them, we will record that patient as lost to follow up but include a sensitivity analysis including these patients as having died.

If the patient dies in hospital, the date and cause of death is recorded in the chart and will be recorded. If the patient dies outside of hospital, this may not be readily apparent. However, survival is more common than death and in-hospital death is more common than out of hospital death.

Section 8 – Assessment of Safety

8.1 Specification of safety parameters

1) Discontinuation of study drug

2) Adverse events

8.2 The methods and timing for assessing, recording, and analysing safety parameters.

Discontinuation of study drug (day 28):

At the day 28 in-person or remote visit we will ask about adherence to the study drug and completion of the prescribed course.

Other adverse events:

Serious events will be reviewed and classified by the site PIs. Severity will be classified using a standard set of criteria for grading adverse events. The relationship between the event and study drug and whether or not the event is expected will be assessed from the product monograph (blinded to assignment).

All deaths and serious adverse events will be notified to the NPI and considered by the data safety monitoring board and will be included in the final analysis. All unforeseen events will be reviewed during regular teleconferences of site investigators as well as reported.

Any events that are unexpected (in terms of severity or frequency), that can reasonably be attributed to the drug under study and that may expose other subjects to harm will be reported.

SAE/Safety/SUSAR events refers to problems, in general, to include any incident, experience, or outcome (including adverse events) that meets ALL the following criteria:

1. **Unexpected**
   In terms of nature, severity or frequency of the problem as described in the monograph
2. **Related or possibly related to participation in the research**
   Possibly related means there is a reasonable possibility that the problem may have been caused by the procedures involved in the research; and
3. **Risk of harm**

Suggests that the research places participants or others at a greater risk of harm (including physical, psychological, economic, or social harm) than was previously known or recognized.

Reporting Timeline for SAE/Safety/SUSAR Events:

1. Urgent Reporting: All problems involving local deaths, whether related or not, should be reported immediately – within 24 hours after first knowledge by the local PI.

2. Expedited Reporting: All other problems must be reported as soon as possible but not later than 7 calendar days after first knowledge by the local investigator.

8.3 Procedures for eliciting reports of and for recording and reporting adverse event and intercurrent illnesses.

This is a pragmatic trial using a drug which is approved in Canada and frequently used. Eligible participants must tolerate 7-10 days of a planned 14 days of oral vancomycin standard of care treatment for their current CDI (or first recurrence of CDI) in order to participate in this extended (2 week) vancomycin vs placebo dose tapering study. We are seeing these patients in-person or remotely (phone/video consultation) at the completion of exposure to study drug. Outside of scheduled visits, we have asked patients to contact us if they believe they have had an adverse event. We also provide a wallet information card for the patients so they can show other doctors and healthcare practitioners which gives contact information for the study for the purposes of questions and reporting. We will also review available clinical documentation as appropriate.

8.4 The type and duration of the follow-up of subjects after adverse events.

Care is provided by the clinical teams and management of adverse events will be as clinically appropriate.

Section 9 – Statistics

9.1 A description of the statistical methods to be employed, including timing of any planned interim analysis.

Primary Outcome:

An intention-to-treat analysis will be used for the primary outcome (recurrence at 56 days).

We will use a Bayesian generalized linear model (binomial family, log link; adjusting for treatment assignment and stratification) to estimate the relative risk, 95% Credible Intervals, and probability of superiority (probability that log relative risk is <0). We will use 4 chains of 50,000 Markov Chain Monte Carlo simulations and verify convergence using the Gelman–Rubin convergence diagnostic.

The relative risk will be used to estimate the absolute risk reduction from the control event rate (with 95% CrI) and subsequently the "number needed to treat (NNT)".

Pre-specified subgroup analyses will be performed based on: initial episode vs. first recurrence; ELISA result; NAP1 type of *C. difficile* of initial/recurrent isolates; efficacy of intervention based on PCR result at day 14 of initial therapy. We will also conduct pre-specified subgroup analyses for: age; biological sex; solid organ or hematologic transplant patients; malignancy; inflammatory bowel disease; PPI use; by first recurrence at enrollment; and nosocomial vs. community acquired.

Secondary Outcomes:

For each binary secondary outcome, we will use a Bayesian generalized linear model (binomial family, log link; adjusting for treatment assignment and stratification) to estimate the relative risk, 95% Credible Intervals, and probability of superiority (probability that log relative risk is <0). We will use 4 chains of 50,000 Markov Chain Monte Carlo simulations and verify convergence using the Gelman–Rubin convergence diagnostic.

The exit CDiff32 scores will be compared by ANCOVA after adjusting for any available baseline scores.

Sensitivity Analyses:

- We will conduct a per protocol analysis for the primary outcome (see 9.7).
- We will also present a time-to-event analysis using a Bayesian parametric survival model (Weibull survival model) adjusting for treatment assignment and stratification. Patients will be censored at death or at the end of follow-up (Days 56 and 90 respectively).
- Standard Kaplan Meier curves will be presented for recurrence.

Interim analyses:

Interim analysis will be performed when 25% and 50% of subjects have completed the day 56 follow up (including those who had the primary outcome prior to day 56) and presented to a Data Safety Monitoring Board (see 3.3). The analysis and presentation will be peformed blinded.

Simulations were conducted with adaptR in the R environment (version 4.2). 10,000 simulated trials were conducted under both the null and alternative hypotheses with a non-informative [beta(1,1)] prior. The estimated trial wide type 1 error under these assumptions is 5.1% and the estimated power is 72.6%.

The trial will be stopped for superiority if the posterior probability of superiority exceeds the probability threshold (99% @ 125, 97.5% @ 250 and 97.5% at full sample size).

The trial will be stopped for clinical futility if the posterior probability of at least a 4% absolute risk reduction (NNT 25; felt to be the minimum clinically meaningful difference) is below 15%.

9.2 The number of subjects planned to be enrolled.

The estimated number of CDI cases available has been based on fiscal year 2016 data: total of 1770 per year.

The risk of recurrence is estimated at 25%.[8,9,14,16] We wish to demonstrate that an initial tapering regimen is associated with an absolute decrease in the risk of relapse of at least 10% (number needed to treat of 10) which would be similar to than the effect seen within 40 days in the fidaxomicin trial.[14] This estimate accounts for our longer period of follow up and will allow some flexibility in the actual recurrence rate found in our control arm. With frequentist 80% power and a type 1 error of 5%, this would require **248 patients to complete follow up in each arm** (total 496) which would be 552 in the case of 10% loss to follow up.

In 2022 the IDSA recommended fidaxomicin as first line therapy for *C. difficile*. This had the effect of simultaneously (a) increasing the potential financial relevance of the TAPER-V strategy and (b) threatening the viability of the trial to continue to recruit if Canadian guidelines followed. The Canadian guidelines have not yet changed.

It was therefore decided to revise the analytic plan well in advance of the first interim analysis. We chose a Bayesian adaptive design to facilitate earlier meaningful comparisons while preserving overall type 1 error at ~5%. Planned analyses will occur at 125, 250 and 500 patients with an estimation of the probability of superiority and futility at each analysis (see 9.1).

Recruitment, loss to follow up and compliance are discussed below.

Recruitment rates and loss to follow-up

Based on our previously published McGill CDI cohort, we estimate 10% will be ineligible and 5-10% will die within 90 days of the initial diagnosis[8]. Of the remaining 80%, we estimate 50%

recruitment although, in general, patients with CDI are highly motivated to avoid subsequent episodes and this estimate is more conservative than the recruitment rate for our centres who participated in the fidaxomicin trial. We estimate 90% complete follow up on those who survive. We estimated a loss to follow up of 10% to ensure we could still complete the study, as designed, within the allocated time and budget even if there were unforeseen challenges. However, loss to follow up at day 40 in the fidaxomicin trial was less than 5% and our method of follow up is varied and flexible, involves dedicated study personnel, and is based on patient preference (options include phone, text message, email, and in person). With these estimates designed to ensure we can succeed in this trial; we would estimate recruiting approximately 566 patients per year and could complete the study with an estimated total duration of 24 months including adjudication and analysis. None of the participating centres will run competing studies.

Compliance

Compliance will likely be high. In a large *C. difficile* RCT, which included 3 of our study hospitals,[14] the average adherence to vancomycin in the intention-to-treat analysis was 91.4% and in per-protocol 96.1%. Patients with *C. difficile* are generally highly motivated to avoid subsequent episodes and we have purposefully chosen a regimen which minimizes pill burden.

9.3 The level of significance to be used.

The trial wide type 1 error is estimated from simulation at 5.1%. We will report both the probability of superiority (log risk ratio <0) as well as the point estimate of the risk ratio and the 95% Credible Interval.

9.4 Criteria for the termination of the trial.

The study will be terminated early based on stopping rules above.

Alternatively, if completion of the trial appears to be infeasible due to (a) disruption due to COVID-19, (b) loss or depletion of funding, (c) change in Canadian treatment guidelines favoring fidaxomicin or another agent other than vancomycin or (d) lack of recruitment defined as fewer than 10 patients recruited per month for 6 months, the trial may be stopped. If this occurs, we will report out the probability of superiority for the primary outcome effect size.

9.5 Procedure for accounting for missing, unused, and spurious data.

Missing data will be imputed by multiple imputation. There is not expected to be unused data. Spurious data, if identified, will be included and replaced by multiple imputation as part of a sensitivity analysis.

9.6 Procedures for reporting any deviation(s) from the original statistical plan (any deviation(s) from the original statistical plan should be described and justified in protocol and/or in the final report, as appropriate).

Changes to the statistical plan will be reported as such in any subsequent publication. This means that the original statistical plan will be reported with justification for changes made in the peer reviewed manuscript.

Currently, the only change which will require reporting is the switch to a Bayesian informed design as described in sections 9.1 and 9.2.

9.7 The selection of subjects to be included in the analyses (e.g., all randomized subjects, all dosed subjects, all eligible subjects, evaluable subjects).

We will perform analyses of the primary outcome both in the intention to treat and per-protocol populations.

Intention to treat:

Patients will be analyzed according to the group they were randomized.

Per protocol:

All patients who receive all 14 days of study medication if no recurrence prior to 28 days (up to 3 missed pills allowed). If recurrence has occurred prior to 28 days, receipt of study medication up until the time of recurrence would constitute "per protocol" (no missed pills allowed). Deviations from protocol (e.g., off study vancomycin exposure) will not be included in the per-protocol analysis if they occurred before day 28. Participants who receive open label vancomycin before day 28 (unless it is being given at treatment doses for a recurrence) in the control group are excluded (cross-over). An additional more stringent per protocol analysis will exclude all patients who received off-study vancomycin beyond day 28 (unless it was given for treatment of a recurrence).

Sensitivity analysis for patients who are lost to follow up will assume they have had the primary outcome and/or death after final contact.

Section 10 – Direct Access to Source Data/Documents

All participating Canadian sites will agree to permit confidential access to source data and documents for the purposes of trial-related monitoring, audits, ethics board review, and Health Canada regulatory inspections.

Section 11 – Quality Control and Quality Assurance

Audits of a random selection of case report forms for quality and accuracy will happen quarterly or every 10 patients recruited, whichever is longer. If deficiencies are identified, a larger audit will be conducted, retraining of study personnel will be conducted, and deficiencies corrected.

Section 12 – Ethics

This study will be conducted in accordance with the principles of Good Clinical Practice and the Declaration of Helsinki. All study sites will obtain approval from their ethics review boards and follow local policies and standard operating procedures.

Section 13 – Data Handling and Record Keeping

Consent forms will be stored for each patient in a locked cabinet and will contain the study ID. All other trial data will be securely housed at McGill University on our trials server system. Specifically, an instance of the CloudTrials platform (invented by Dr. Lee and housed securely at McGill) will host the trial database. CloudTrials has user access controls such that only verified accounts have access to the application. CloudTrials allows each aspect of the study from screening eligibility, entering baseline data, randomization, entering follow up data (including patient reported outcomes), and study tracking at the individual (coded) and site levels as well as overall enrollment/completion/loss to follow up.

CloudTrials does this while maintaining central anonymity – meaning that each site only has access to their patients and their personal identifying data. The database keeps personal identifying data in a separate and fully encrypted table with decryption keys tied to the individual account. The encryption uses an algorithm which is not easily compromised even with modern cryptographic techniques. This means that even if the database itself were compromised, none of the identifying data would be. At the end of the study, all data will become coded with the encrypted personal information removed and only the study ID retained. Each site will have linked consent forms to that study ID.

CloudTrials uses HTTPS secure transmission and the webserver itself is housed within a the McGill university network. That network is behind a firewall contained within the McGill network which itself is behind a firewall and reverse proxy. The backend database is housed on a separate machine and is a password secured oracle SQL enterprise database. This network infrastructure has supported several randomized controlled trials.

After study completion, study data will be archived for 15 years as per Health Canada regulations.

Section 14 – Financing and Insurance

This study is CIHR funded and investigator-initiated and derives no income from industry sources.

Each participating center will make sure that their research institute has the correct insurance policies for conducting investigator-initiated randomized controlled trials and that each participating physician-investigator has appropriate malpractice insurance for the Province in which they practice.

Section 15 – Publication Policy

This work will be published regardless of the result. Investigators who meet the ICJME criteria for authorship will have the opportunity to be co-authors on the manuscripts.

REFERENCES

1. Loo VG, Davis I, Embil J, et al. Association of Medical Microbiology and Infectious Disease Canada treatment practice guidelines for Clostridium difficile infection. Official Journal of the Association of Medical Microbiology and Infectious Disease Canada 2018;3:71-92.
2. Leffler DA, Lamont JT. Clostridium difficile Infection. New England Journal of Medicine 2015;372:1539-48.
3. Lessa FC, Gould CV, McDonald LC. Current Status of Clostridium difficile Infection Epidemiology. Clinical Infectious Diseases 2012;55:S65-S70.
4. Levy AR, Szabo SM, Lozano-Ortega G, et al. Incidence and Costs of Clostridium difficile Infections in Canada. Open forum infectious diseases 2015;2:ofv076.
5. The L. A new approach to treating infection. The Lancet;391:714.
6. Dobson G, Hickey C, Trinder J. Clostridium difficile colitis causing toxic megacolon, severe sepsis and multiple organ dysfunction syndrome. Intensive Care Medicine 2003;29:1030-.
7. Mylonakis E, Ryan ET, Calderwood SB. Clostridium difficile–associated diarrhea: A review. Archives of internal medicine 2001;161:525-33.
8. McDonald EG, Milligan J, Frenette C, Lee TC. Continuous Proton Pump Inhibitor Therapy and the Associated Risk of Recurrent Clostridium difficile Infection. JAMA internal medicine 2015;175:784-91.
9. Sheitoyan-Pesant C, Abou Chakra CN, Pepin J, Marcil-Heguy A, Nault V, Valiquette L. Clinical and Healthcare Burden of Multiple Recurrences of Clostridium difficile Infection. Clinical infectious diseases : an official publication of the Infectious Diseases Society of America 2016;62:574-80.
10. Rodrigues R, Barber GE, Ananthakrishnan AN. A Comprehensive Study of Costs Associated With Recurrent Clostridium difficile Infection. Infection control and hospital epidemiology : the official journal of the Society of Hospital Epidemiologists of America 2017;38:196-202.
11. Olsen MA, Yan Y, Reske KA, Zilberberg MD, Dubberke ER. Recurrent Clostridium difficile infection is associated with increased mortality. Clinical microbiology and infection : the official

publication of the European Society of Clinical Microbiology and Infectious Diseases 2015;21:164-70.
12. Wenisch C, Parschalk B, Hasenhündl M, Hirschl AM, Graninger W. Comparison of Vancomycin, Teicoplanin, Metronidazole, and Fusidic Acid for the Treatment of Clostridium difficile—Associated Diarrhea. Clinical Infectious Diseases 1996;22:813-8.
13. Zar FA, Bakkanagari SR, Moorthi KMLST, Davis MB. A Comparison of Vancomycin and Metronidazole for the Treatment of Clostridium difficile–Associated Diarrhea, Stratified by Disease Severity. Clinical Infectious Diseases 2007;45:302-7.
14. Louie TJ, Miller MA, Mullane KM, et al. Fidaxomicin versus vancomycin for Clostridium difficile infection. The New England journal of medicine 2011;364:422-31.
15. McDonald LC, Gerding DN, Johnson S, et al. Clinical Practice Guidelines for Clostridium difficile Infection in Adults and Children: 2017 Update by the Infectious Diseases Society of America (IDSA) and Society for Healthcare Epidemiology of America (SHEA). Clinical Infectious Diseases 2018:cix1085-cix.
16. Wilcox MH, Gerding DN, Poxton IR, et al. Bezlotoxumab for Prevention of Recurrent Clostridium difficile Infection. New England Journal of Medicine 2017;376:305-17.
17. Escobar GJ, Baker JM, Kipnis P, et al. Prediction of Recurrent Clostridium Difficile Infection Using Comprehensive Electronic Medical Records in an Integrated Healthcare Delivery System. Infection Control & Hospital Epidemiology 2017:1-8.
18. Ma GK, Brensinger CM, Wu Q, Lewis JD. Increasing Incidence of Multiply Recurrent Clostridium difficile Infection in the United States: A Cohort Study. Annals of internal medicine 2017;167:152-8.
19. Marrie TJ, Faulkner RS, Badley BW, Hartlen MR, Comeau SA, Miller HR. Pseudomembranous colitis: isolation of two species of cytotoxic clostridia and successful treatment with vancomycin. Canadian Medical Association journal 1978;119:1058-60.
20. McFarland LV, Elmer GW, Surawicz CM. Breaking the cycle: treatment strategies for 163 cases of recurrent Clostridium difficile disease. Am J Gastroenterol 2002;97:1769-75.
21. Crowther GS, Chilton CH, Longshaw C, et al. Efficacy of vancomycin extended-dosing regimens for treatment of simulated Clostridium difficile infection within an in vitro human gut model. The Journal of antimicrobial chemotherapy 2016;71:986-91.
22. Assessment Report: Dificlir (Fidaxomicin). European Medicines Agency, 2011. (Accessed January 1, 2018, at http://www.ema.europa.eu/docs/en_GB/document_library/EPAR_-_Public_assessment_report/human/002087/WC500119707.pdf.)
23. Cohen SH, Gerding DN, Johnson S, et al. Clinical practice guidelines for Clostridium difficile infection in adults: 2010 update by the society for healthcare epidemiology of America (SHEA) and the infectious diseases society of America (IDSA). Infection control and hospital epidemiology : the official journal of the Society of Hospital Epidemiologists of America 2010;31:431-55.
24. DeMets DL, Lan KK. Interim analysis: the alpha spending function approach. Statistics in medicine 1994;13:1341-52; discussion 53-6.

TAPER-V Statistical Analysis Plan

Section 1: Administrative Information

Table 1. Study information

**Date**	April 10, 2023
**Study Title**	Initial Vancomycin Taper for the Prevention of Recurrent Clostridium Difficile Infection (TAPER-V)
**Study Registration Number**	NCT04138706
**SAP Version Number**	Version 1.2 (10/APR/2023)<br>Version 1.1 (12/DEC/2022)<br>Version 1.0 (02/NOV/2020)
**Protocol Version and Date**	Version 3.4 (3/APR/2023)
**Trial Statistics**	*James M. Brophy and Todd C. Lee*
**Trial Principal Investigators**	*Todd C. Lee and Emily G. McDonald*
**SAP Author(s)**	*Todd C. Lee and Emily G. McDonald*<br>*Revised with James M. Brophy*

1.1 Revision Control

SAP Version	Protocol Version	Section numbers changed	Description of Changes	Date Changed
1.0	3.1	--	--	11/02/2020
1.1	3.3	1.1, 3.3,3.4,4.1,6.1, 6.2,and 6.4	Described below	12/12/2022
1.2	3.4	1.0, 1.1, 2.3.2, 4.4	Addition of Dr. Brophy;<br>Addition of secondary outcome at day 38 to compare to fidaxomicin;<br>Clarification of Per Protocol	10/4/2023

The initial statistical analysis plan called for two interim looks at 125 and 250 patients using O'Brien-Flemming alpha spending boundaries. However, by 2022, the Infectious Diseases Society of America (IDSA) recommended fidaxomicin as first line therapy for *C. difficile*.[1] This had the effect of simultaneously (a) increasing the potential financial relevance of the TAPER-V strategy and (b) threatening the viability of the trial to continue to recruit if Canadian guidelines followed. It was therefore decided to revise the analytic plan well in advance of the first interim analysis. At the time of this revision, approximately 65 patients had been recruited to the trial. We chose a Bayesian adaptive design to facilitate earlier meaningful comparisons while preserving overall type 1 error at ~5%. Specifically, the Bayesian approach allows for earlier conclusions optimizing resource allocation and maximizing power while assuring the minimum number of patients will receive an inferior treatment. Planned analyses will occur at 125, 250 and 500 patients with an estimation of the probability of superiority at each analysis. The relevant sections have been updated with the original text retained for comparison.

1.2 Roles and responsibilities

**Name**	**Role**	**Institution**
Todd C. Lee	*Principal Investigator*	*Research Institute- McGill University Health Centre (RI-MUHC)*
Emily McDonald	*Co-Principal Investigator*	*RI-MUHC*
James M. Brophy and Todd C. Lee	*Trial Statistics*	*RI-MUHC*

1.3 Contributions

Todd Lee and Emily McDonald developed the statistical analysis plan (SAP) based on the analyses set out in the trial protocol and considering any logistical challenges introduced by COVID-19 and the potential for changes in national guidelines for CDI in Canada which might lead to premature termination of the study.

1.4 Abbreviations and Definitions

TAPER-V

CDI - *C. difficile* infection.

PCR - polymerase chain reaction

RI-MUHC Research Institute of the McGill University Health Centre

Section 2: Introduction

2.1 Background and Rationale

The rationale for TAPER-V is well described in the core protocol. Briefly, we hypothesized that the risk for recurrence was highest within the first weeks following discontinuation of vancomycin therapy and that the introduction of a 2-week tapering regimen of vancomycin (125mg PO BID x 7 days followed by 125mg PO DIE x 7 days) would reduce the rate of recurrence within 56 days. We standardized initial treatment to 14 days based on Canadian guidelines.[2] A placebo control was chosen to prevent bias in patient and clinician interpretation of any lingering GI symptoms. The taper dosing regimen for vancomycin was chosen because it matched the middle steps of the most common 6-week taper which was used in Canada.

2.2 Objectives

To evaluate whether a 2-week vancomycin tapering regimen, given at the end of 14 days of initial vancomycin therapy, will reduce CDI recurrence within 56 days.

2.3 Study Outcome Measures

Assessment of outcome measures will be through a combination of in person and virtual visits as well as by self-report. As necessary, medical records will be consulted.

2.3.1 Primary Outcome Measures

Primary outcome is defined as the proportion of patients who have a recurrence within 56 days of the initial dose of vancomycin (Day 1).

Definition: CDI recurrence will be defined by three or more diarrheal stools/24-hour period coupled with a positive PCR for toxin gene or and/or detection of toxin by EIA or CCA and administration of treatment. However, to avoid missing severe recurrences: for cases of ileus, toxic megacolon, or pseudomembranous colitis on colonoscopy the test result will be used in the absence of three or more stools.

Patients reporting diarrhea will be brought in for an in-person interview or booked for a telephone/video outpatient visit and investigated as appropriate clinically without unblinding. Additionally, patients and/or their proxy will be instructed to contact the study team if they believe they are having a recurrence between contacts. Patients will be able to come be assessed for potential relapse by infectious diseases physicians at each site (who may or may not be a part of the study) or could see their usual doctors. Recurrence will be assessed by clinical record review (chart, laboratory, pharmacy records) and any direct patient interview. Blinded case summaries will be reviewed in duplicate with disagreement resolved by consensus.

2.3.2 Secondary Outcome Measures

- Late recurrence out to 90 days: as defined above
- Use of fidaxomicin, colectomy, or fecal microbiota transplantation within 90 days: based on patient report and review of medical records
- All-cause death within 90 days: If a patient misses their day 28 or day 56 in-person follow up, we will initially review their hospital file to see if they have died. If this does not show death, we will contact the patient (or their proxy) by telephone to determine why they missed the follow up and reschedule it as required. If we cannot reach the patient or their proxy, and the patient has not been replying to the email/text/phone surveys we will search the obituaries. After day 56 we will use the survey responses as proof of life. If there is no response to the day 90 survey by day 95, we will first check the hospital file to see if they have died. Then, if vital status is not clear, we will search the obituaries. Then if vital status is still not clear, we will attempt to reach the patient by telephone and registered mail. If we cannot reach the patient, we will record that patient as lost to follow up but include a sensitivity analysis including these patients as having died.
- *C. difficile* associated quality of life at day 56: At day 56 patients will be asked to provide a measure of self-reported quality of life using the Cdiff32 score which was developed and validated to evaluate health-related quality of life in patients with *C. difficile*.[3] Each item in the Cdiff32 Quality of Life Questionnaire is scored between 0 (worst quality of life) to 100 (best quality of life).
- Emergency Room visit within 90 days: Within 90 days post enrolment we will access the medical record and we will also analyze patient emails/text surveys for all emergency department visits and will record the date of the first emergency department visit. Patient charts will also be flagged for immediate review should they visit the emergency room or be admitted to study centres to assess for the primary and other secondary outcomes. With explicit written patient consent, medical records from outside hospitals will be also requested for review if they report presenting elsewhere.
- Readmission to hospital within 90 days: Within 90 days post enrolment we will access the medical record and we will also analyze patient emails/text surveys for all hospital readmissions and will record the date of the first hospital readmission. Patient charts will also be flagged for immediate review should they be readmitted to study centres to assess the primary and other secondary outcomes. With explicit written patient consent, medical records from outside hospitals will also be requested for review if they report presenting elsewhere. Patients who are readmitted to the hospital will not be counted as having had an emergency room visit for that readmission.
- Discontinuation of study drug at 28 days: At the day 28 visit we will inquire about adherence and the quantity of pills remaining.

- Receipt of non-study antibiotics within 90 days: Exposure to antibiotics for infections other than *C. difficile* may occur. We will ask patients to contact us if this occurs so that the appropriate data can be recorded to account for these effects outside of the study. We also record this on the weekly surveys.
- Use of vancomycin secondary prophylaxis within 90 days: Exposure to antibiotics for other infections may lead to the receipt of vancomycin secondary prophylaxis in that context. We will ask patients to contact us if this occurs so that the appropriate data can be recorded to account for these effects outside of the study. If vancomycin secondary prophylaxis has occurred prior to day 28, the patient will stop the study drug to avoid excess vancomycin exposure, and this will be recorded. We also record this in the weekly surveys.
- Economic analysis: Economic analysis will be carried out from the perspective of the McGill University Health Centre (MUHC) following established guidelines and will be generalized to the rest of Canada. We will estimate the average cost per patient of using the interventions under study routinely in patients with *C. difficile* diarrhea. We will also gather information on the cost of health services use (hospitalizations and other interventions such as colectomy) following this intervention to determine the budget impact compared with routine practice. If the intervention proves efficacious, we will carry out a cost-benefit or cost-effectiveness analysis to determine the incremental cost per case of *C. difficile* diarrhea avoided. The base case will be the placebo group, to which the treatment will be compared. Further, we will carry out a cost utility analysis to report the incremental cost per unit increase in quality of life as measured by the Cdiff32 score.
- Safety & tolerability of the vancomycin extension/taper treatment period (i.e., days 15-28 inclusive): We will create a survey at day 28 asking participants to communicate any side effects from the study medication, with a section for detailing any adverse reactions.

Version 1.2

- To facilitate direct comparison to the fidaxomicin registrational trials, an additional secondary outcome will be reported at day 38.

Section 3: Study Methods

3.1 Trial Design

Randomized placebo-controlled trial with 1:1 allocation to placebo or intervention. The initial treatment is standardized to vancomycin 125mg PO QID for 14 days (with allowances for higher initial doses and combination therapy with metronidazole due to severity of illness).

Intervention: Vancomycin capsules 125mg PO BID x 7 days then 125mg PO DIE x 7 days

Control: Placebo capsules PO BID x 7 days then 125mg PO DIE x 7 days

Figure 1 Schematic of Trial

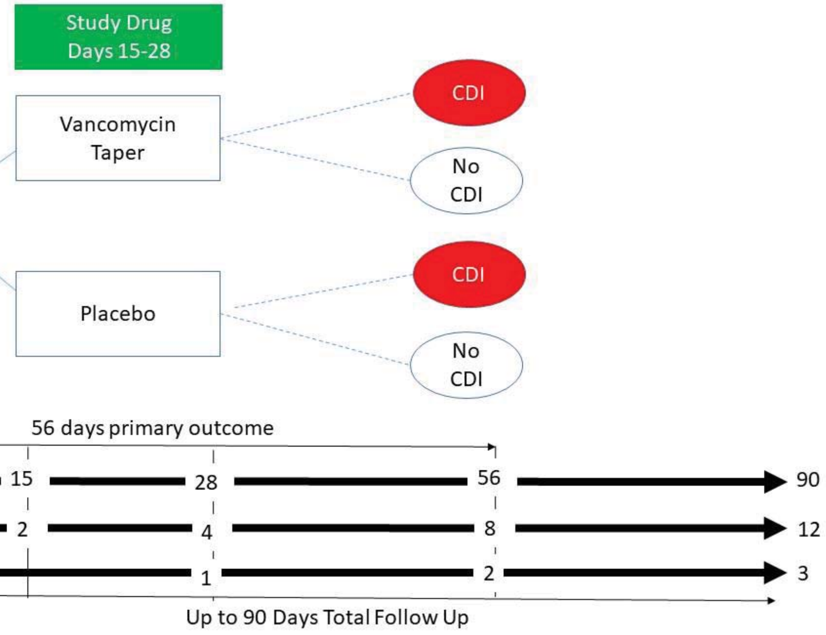

Additional details are contained within the protocol.

Subjects are randomized at the time of enrolment (days 7-10) and receive the treatment to start on Day 15. Follow-up is for 90 days starting on the first day of initial vancomycin treatment.

3.2 Randomization

3.2.1 Randomization Procedures

As soon as consent is obtained, and the subject's eligibility is confirmed, they are randomized via permuted block randomization. The randomization table was created using permuted blocks of random sizes (2, 4, 6, 8), stratified by first episode or first recurrence and stored within the trial database. Study investigators, research assistants, analysts, and subjects will be blinded.

Only the research pharmacy will be aware of the assignment.

3.2.2 Masking Procedures

Participants will be provided masked study medicine, either hand delivered or shipped overnight by courier (e.g., Purolator). The intervention vs. placebo are not 100% physically

identical; however, they are extremely similar, and participants have been informed that study drugs may not match those they were receiving in the hospital. In reality, this is also true for vancomycin capsules where those used in the hospital and those dispensed in the community often differ because of differing suppliers. A “treatment guess” is conducted on Day 28 to evaluate whether subjects could identify their assignment; however, even if imperfect, the desired effect of blinding the treating clinicians and investigators remains important.

3.3 Sample Size

Version 1

The risk of recurrence is estimated at 25%. We wish to demonstrate that an initial tapering regimen is associated with an absolute decrease in the risk of relapse of at least 10% (number needed to treat 10) which would be similar to the effect seen within 40 days in the fidaxomicin trial.4 This estimate accounts for our longer period of follow up and will allow some flexibility in the actual recurrence rate found in our control arm. With 80% power and a type 1 error of 5%, this would require 248 patients to complete follow up in each arm (total 496; rounded to 500) which with 10% loss to follow up would equal 552.

Version 1.1

The total maximum sample size of 500 is unchanged for the Bayesian analysis. Based on 50,000 simulated trials under the above effect size assumptions in adaptR[5], 23% of simulations require the full sample size; however, the mean sample size is 341 (SD 154) and the median is 250 (IQR 250-500).

3.4 Statistical Interim analyses and stopping guidance (if applicable)

All interim analyses will be performed blinded, and a blinded report will be presented to the data safety monitoring board. If deemed necessary, a fully unblinded report can be requested by the chair of the data safety monitoring board in writing.

Version 1

Blinded interim analysis will be performed when 25% and 50% of subjects have completed the day 56 follow up (including those who had the primary outcome prior to day 56) and presented to a Data Safety Monitoring Board. We will use pre-specified rules for stopping the study while accounting for appropriate alpha-spending using the O’Brien-Fleming approach. Specifically, the first interim will use a p-value of 0.00001473 and the second interim will use a p-value of 0.003036.

If completion of the trial appears to be infeasible due to (a) disruption due to COVID-19 or other external factors, (b) loss or depletion of funding, (c) change in Canadian treatment guidelines favoring fidaxomicin or another agent other than vancomycin or (d) lack of recruitment defined as fewer than 10 patients recruited per month for 6 months, the trial

may be stopped. If this occurs, the trial could be underpowered. We will use all of the remaining alpha for the comparison.

Version 1.1

A non-informative beta(1,1) prior was chosen. Analyses will be conducted when 125, 250, and 500 patients have completed follow up. The trial will be stopped if:

- For superiority if the posterior probability of superiority exceeds the threshold (99% @ 125, 97.5% @ 250 and 97.5% at full sample size).
- For futility if the posterior probability of at least a 4% absolute risk reduction (NNT 25; felt to be the minimum clinically meaningful difference) is below 15%.

Simulations were conducted with adaptR[5] in the R environment (version 4.2). 50,000 simulated trials were conducted under both the null and alternative hypotheses. The estimated trial wide type 1 error under these assumptions is 5.1% and the estimated power is 72.6%.

If completion of the trial appears to be infeasible due to (a) disruption due to COVID-19 or other external factors, (b) loss or depletion of funding, (c) change in Canadian treatment guidelines favoring fidaxomicin or another agent other than vancomycin which impacts recruitment or (d) lack of recruitment defined as fewer than 10 patients recruited per month for 6 months, the trial may be stopped. If this occurs, the trial could be underpowered; however, we will report out the probability of superiority for the primary outcome effect size.

3.5 Timing of final analysis

Once all enrolled subjects have completed 90 days of follow up, we will clean and lock the database. The data analysis will be performed blinded (drug A vs. B). The primary manuscript will include the primary outcome and all secondary outcomes except the quality of life and economic analysis which would be the focus of its own manuscript.

3.6 Timing of outcome assessment

Day 1 is the first day of initial vancomycin treatment. Patients are screened between days 7 and 10 to select subjects who have had a good clinical response. They complete their vancomycin on Day 14. From Days 15 to 28 they receive study drugs. From days 15 through 56 subjects receive weekly email/text/phone calls to determine whether or not they have had an outcome. From days 57 through 90, these contacts are made every 2 weeks. Affirmative responses are followed up for additional details including dates and times.

The primary outcome is assessed by day 56. Adverse events and early vancomycin are assessed out to day 28 (+/- 3 days). Quality of life is assessed at day 56 (+/- 3 days). Other secondary outcomes are assessed out to day 90 (+/- 7 days).

Section 4: Statistical Principles

4.1 Confidence Intervals and P-values

Version 1

We will use 95% confidence intervals and 2-sided p-values while maintaining the total alpha spending for the primary outcome at 0.05. Alpha spending is discussed in section 3.4.

There will be no planned adjustments for the multiple analyses of the secondary outcomes, but we will provide appropriate caution around their interpretation.

Version 1.1

We will report the point estimate for the posterior probability relative risk, 95% credible intervals, and probability of superiority (risk ratio <1).

There will be no planned adjustments for the multiple analyses of the secondary outcomes.

4.2 Adherence and protocol deviations

Adherence to the intervention: This will be assessed based on the day 28 interview. The patient is asked how many pills are remaining and whether they missed any doses.

The following will be considered major protocol deviations (MPD):

1. Receipt of open label vancomycin before day 28 **unless** it is being given at treatment doses for a recurrence.

We will assess major protocol deviations with the weekly surveys and on day 28.

4.3 Approach to missing data

We believe that our close follow up will allow us to minimize missing outcomes data. Patients who are lost before day 56 will be replaced to achieve the total desired sample size. If we lose patients to follow up after day 56, we will censor them at the last known follow-up time. A sensitivity analysis will count them as failures.

4.4 Analysis populations

Intention to Treat

We will use the intention to treat approach where subjects will be analyzed according to the group they were randomized.

Per Protocol

The per-protocol analysis population: all patients who receive all 14 days of study medication if no recurrence prior to 28 days (up to 3 missed pills allowed). If recurrence has occurred prior to 28 days, receipt of study medication up until the time of recurrence would constitute "per protocol" (no missed pills allowed). Major protocol deviations in the control group (as above) are excluded (cross-over).

Version 1.2

An additional more stringent per protocol analysis will exclude all patients who received off-study vancomycin beyond day 28 (unless it was given for treatment of a recurrence).

Safety Analysis Population

The safety analysis population: will include all participants who took at least one dose of study drug.

Section 5: Trial Population

5.1 Eligibility

The eligibility criteria are described in the protocol and clinicaltrials.gov

5.2 Withdrawal/Follow-up

The main reason for discontinuation would be allergy or adverse reactions to the study drug. Equally, the patient can withdraw at any time. Subjects who withdraw for the study or who are lost (before day 56) will be replaced to achieve the necessary sample size.

If a patient decides to withdraw, we will ask them to keep the study data accrued to date for the purposes of scientific integrity. We will also ask them if they would consider completing the 90-day follow-up period electronically.

5.3 Baseline Patient Characteristics

Baseline information will be determined from the screening and enrollment process via chart review and patient interview. Table 1 will contain the demographic data with n(%), means (SD), and medians (IQR) as appropriate. No statistical comparisons will be presented between groups in Table 1.

Section 6: Analysis

6.1 Statistical analysis of primary and secondary outcomes

Primary outcome

Version 1

The proportion of patients who meet the primary outcome will be compared by binomial regression adjusting for stratification and the difference will be reported as an absolute risk difference with 95% confidence intervals and the p-value.

Version 1.1

We will use a Bayesian generalized linear model (binomial family, log link; adjusting for treatment assignment and stratification [1st episode vs. 1st recurrence]) to estimate the relative risk, 95% Credible Intervals, and probability of superiority (probability that log relative risk is <0). We will use 4 chains of 50,000 Markov Chain Monte Carlo simulations and verify convergence using the Gelman–Rubin convergence diagnostic. This is planned to be performed in Stata version 17.[6]

The relative risk will be used to estimate the absolute risk reduction from the control event rate (with 95% CrI) and subsequently the "number needed to treat (NNT)" (with 95% CrI).

Secondary outcomes

Version 1

Proportions meeting each secondary outcome will be analyzed by binomial regression adjusting for stratification and the differences will be reported as an absolute risk difference and 95% CI with a p-value.

The exit CDiff32 scores will be compared by ANCOVA after adjusting for any available baseline scores.

Version 1.1

For each binary secondary outcome where there are at least one event in both arms, we will use a Bayesian generalized linear model (binomial family, log link; adjusting for treatment assignment and stratification) to estimate the relative risk, 95% Credible Intervals, and probability of superiority (probability that log relative risk is <0). We will use 4 chains of 50,000 Markov Chain Monte Carlo simulations and verify convergence using the Gelman–Rubin convergence diagnostic.

The exit CDiff32 scores will be compared by ANCOVA after adjusting for any available baseline scores.

6.2 Analysis Methods

Sensitivity analyses

Version 1

- We will conduct a per protocol analysis for the primary outcome.
- We will also present a time-to-event analysis using Cox regression adjusting for treatment assignment and stratification. Patients will be censored at death or at the end of follow-up (Days 56 and 90 respectively).
- Kaplan Meier curves will be presented for recurrence and death.
- See also section 3.4 for how we will handle analysis in the event of premature termination of the study.

Version 1.1

- We will conduct a per protocol analysis for the primary outcome.
- We will also present a time-to-event analysis using a Bayesian parametric survival model (Weibull survival model) adjusting for treatment assignment and stratification. Patients will be censored at death or at the end of follow-up (Days 56 and 90 respectively).
- Standard Kaplan Meier curves will be presented for recurrence.
- See also section 3.4 for how we will handle analysis in the event of premature termination of the study.

Subgroups (presented as forest plots of relative risk for primary outcome)

- Initial episode vs. first recurrence [patients who have already had a recurrence would be at higher risk of subsequent events];
- ELISA result [if available];
- NAP1 type of *C. difficile* of initial/recurrent isolates [if available];
- Efficacy of intervention based on PCR result at day 14 of initial therapy [if available].

We will also conduct pre-specified subgroup analyses for:

- age [categorized Age <65; 65-79;>=80; in general, older patients are considered at higher risk of recurrence and severe disease. They are also at higher risk of death];
- biological sex;
- solid organ or hematologic transplant patients [may be at higher risk of diarrhea because of medications or at higher risk or recurrence due to immunosuppression or antibiotic exposure];
- malignancy [potentially at higher risk due to chemotherapy, antibiotic exposure, or immunosuppression];

- inflammatory bowel disease [IBD patients may have other reasons for diarrhea, may be exposed to antibiotics, and may be at higher risk of complications];
- PPI use [risk factor for recurrence];
- nosocomial vs. community acquired [nosocomial cases may be higher risk].

6.3 Missing Data

Refer to section 4.3.

All attempts will be made to have complete data, particularly for the primary outcome.

6.4 Harms

Harms will include:

1) side effects
2) adverse events and
3) serious adverse events

Adverse events are coded according to severity (severe/serious vs non-severe). Serious adverse events will be coded as 1) not at all, 2) possibly, 3) probably or 4) certainly attributed to the study medication and the clinical details will be provided.

Side effects will be grouped logically by system (e.g., infectious, musculoskeletal, respiratory, etc.).

Secondary outcomes are not considered adverse events unless they are considered due to study medications.

Side Effects:

Version 1.0

We will compare proportions between groups with the chi-square test and report p-values uncorrected for multiplicity.

Version 1.1

Because these are expected to be very rare events given patients must have tolerated 7-10 days of vancomycin on enrollment, we will plan only to compare “any side effect” using a Bayesian generalized linear model (binomial family, log link; adjusting for treatment assignment and stratification) to estimate the relative risk, 95% Credible Intervals, and probability of superiority (probability that log relative risk is <0). We will use 4 chains of 50,000 Markov Chain Monte Carlo simulations and verify convergence using the Gelman–Rubin convergence diagnostic.

The individual side effects will be reported as proportions in patients who took at least one dose of study medication with no statistical comparison.

6.5 Future manuscripts

The primary manuscript will focus on the primary outcome and select secondary outcomes.

Additional manuscripts will address the quality of life and economic analysis as well as any secondary outcomes not presented in the primary manuscript. In the event of a positive study, we will also conduct a secondary analysis to predict those most likely to benefit from treatment using approaches to explore heterogeneity of treatment effect.

Section 7: References

1. Johnson S, Lavergne V, Skinner AM, et al. Clinical Practice Guideline by the Infectious Diseases Society of America (IDSA) and Society for Healthcare Epidemiology of America (SHEA): 2021 Focused Update Guidelines on Management of Clostridioides difficile Infection in Adults. Clinical Infectious Diseases 2021;73(5):e1029–44.
2. Loo VG, Davis I, Embil J, et al. Association of Medical Microbiology and Infectious Disease Canada treatment practice guidelines for Clostridium difficile infection. Journal of the Association of Medical Microbiology and Infectious Disease Canada [Internet] 2018 [cited 2021 Oct 27];Available from: https://jammi.utpjournals.press/doi/abs/10.3138/jammi.2018.02.13
3. Garey KW, Aitken SL, Gschwind L, et al. Development and Validation of a Clostridium difficile Health-related Quality-of-Life Questionnaire. J Clin Gastroenterol 2016;50(8):631–7.
4. Louie TJ, Miller MA, Mullane KM, et al. Fidaxomicin versus vancomycin for Clostridium difficile infection. N Engl J Med 2011;364(5):422–31.
5. Granholm A, Jensen AKG, Lange T, Kaas-Hansen BS. adaptr: an R package for simulating and comparing adaptive clinical trials. Journal of Open Source Software 2022;7(72):4284.
6. Statacorp LP. Bayesian regression models using the bayes prefix in Stata [Internet]. [cited 2022 Dec 12];Available from: https://www.stata.com/features/overview/bayes-prefix/

Supplemental Figures and Tables

Supplementary Table 1. Participating Sites

Site	Lead Investigator(s)	Province
Vancouver General Hospital, University of British Columbia	Theodore S. Steiner	British Columbia
Eastern Health, Memorial University	Peter Daly	Newfoundland
Kingston General Hospital, Queens University	Santiago Perez-Patrigeon	Ontario
Michael Garron Hospital	Jeff E . Powis<br>Christopher E. Kandel	Ontario
Sunnybrook Health Sciences Centre, University of Toronto	Nick Daneman<br>Adrienne Chan	Ontario
The Ottawa Hospital (Ottawa General and Ottawa Civic Hospitals), University of Ottawa	Derek MacFadden<br>Caroline Nott	Ontario
Unity Health – St. Joeseph's Hospital,	Yan Chen	Ontario
Unity Health – St. Michael's Hospital, University of Toronto	Matthew Muller	Ontario
University Health Network (Toronto General and Toronto Western Hospitals), University of Toronto	Bryan Coburn<br>Susy Hota	Ontario
Jewish General Hospital, McGill University	Yves Longtin<br>Ling Kong	Quebec
McGill University Health Centre (Royal Victoria Hospital and Montreal General Hospital), McGill University	Todd C. Lee<br>Emily G. Mcdonald	Quebec
Centre hospitalier universitaire de Sherbrooke (Hôpital Fleurimont and Hôtel-Dieu), Université de Sherbrooke	Louis Valiquette	Quebec

Supplementary Table 2. Collaborators – included as the "TAPER-V Team"

Claire J. Lin<br>Division of Infectious Diseases, Department of Medicine,<br>University of British Columbia, Vancouver, Canada
Malaz Jamal Idris BHSc<br>Division of Infectious Diseases and Respirology, Department of Medicine,<br>University of Ottawa, Ottawa, Canada
Adrienne K. Chan MD MPH<br>Division of Infectious Diseases, Department of Medicine,<br>University of Toronto, Toronto, Canada

Asgar Rishu MBBS MHSc<br>Sunnybrook Research Institute ,<br>University of Toronto, Toronto, Canada
Noelle R. Yee MSc<br>Division of Infectious Diseases, Department of Medicine,<br>University of Toronto, Toronto, Canada
Maria Kulikova DPT<br>Division of Infectious Diseases, Toronto General Hospital Research Institute,<br>University Health Network, Toronto, Canada
Jeff E Powis MD, MSc<br>Department of Medicine,<br>Michael Garron Hospital, Toronto, Canada
Maureen Taylor B.Sc(PA)<br>Department of Medicine,<br>Michael Garron Hospital, Toronto, Canada
James M. Brophy MD PhD<br>Division of Cardiology, Department of Medicine,<br>McGill University, Montreal, Canada

Supplementary Table 3. Data Safety Monitoring Committee

Name	University	Role
Dominik Mertz MD MSc	McMaster University	Chair, Clinical Trialist
Biyue Dai PhD	University of Minnesota	Statistician
Zain Chagla MD	McMaster University	Clinician
Jenine Leal PhD	University of Calgary	Observer
Elissa Rennert May MD MSc	University of Calgary	Observer

Supplementary Table 4. Restricted Mean Survival Time Analysis

Model Summary (difference of Restricted Mean Survival Time)			
	Coefficient	95% Confidence Interval	P-Value
Intercept	31.38	29.9-32.9	<0.001
Treatment	2.19	0.51-3.87	0.01
Stratification (First episode vs. First Relapse)	-1.44	-4.83 to 1.95	0.41
**Model Summary (ratio of Restricted Mean Survival Time)**			
	Exponentiated Coefficient	95% Confidence Interval	P-Value
Intercept	31.38	29.9-32.9	<0.001
Treatment	1.07	1.02-1.13	0.012
Stratification (First episode vs. First Relapse)	0.96	0.86-1.07	0.42

Supplementary Table 5. Per Protocol Analyses and Recurrence Outcomes

Characteristic	Vancomycin	Placebo	Adjusted Relative Risk (95% CrI)	Probability of Superiority
Per Protocol without vancomycin prophylaxis before day 28	(n=122)	(n=116)		
Recurrence of *C. difficile*				
Day 56 - Primary outcome	20 (16.4%)	22 (19.0%)	0.87 (0.49-1.52)	69.8%
Day 38 - Secondary outcome	9 (7.4%)	19 (16.4%)	0.45 (0.20-0.94)	98.4%
Day 90 - Secondary outcome	23 (18.9%)	23 (19.8%)	0.95 (0.56-1.64)	57.8%
Per Protocol without any vancomycin prophylaxis	(n=108)	(n=106)		
Recurrence of *C. difficile*				
Day 56 - Primary outcome	15 (13.9%)	18 (17.0%)	0.81 (0.42-1.53)	74.2%
Day 38 - Secondary outcome	7 (6.5%)	15 (14.2%)	0.44 (0.17-1.02)	97.2%
Day 90 - Secondary outcome	17 (15.7%)	19 (17.9%)	0.88 (0.47-1.59)	66.7%

Supplementary Figure 1. Time to Recurrence

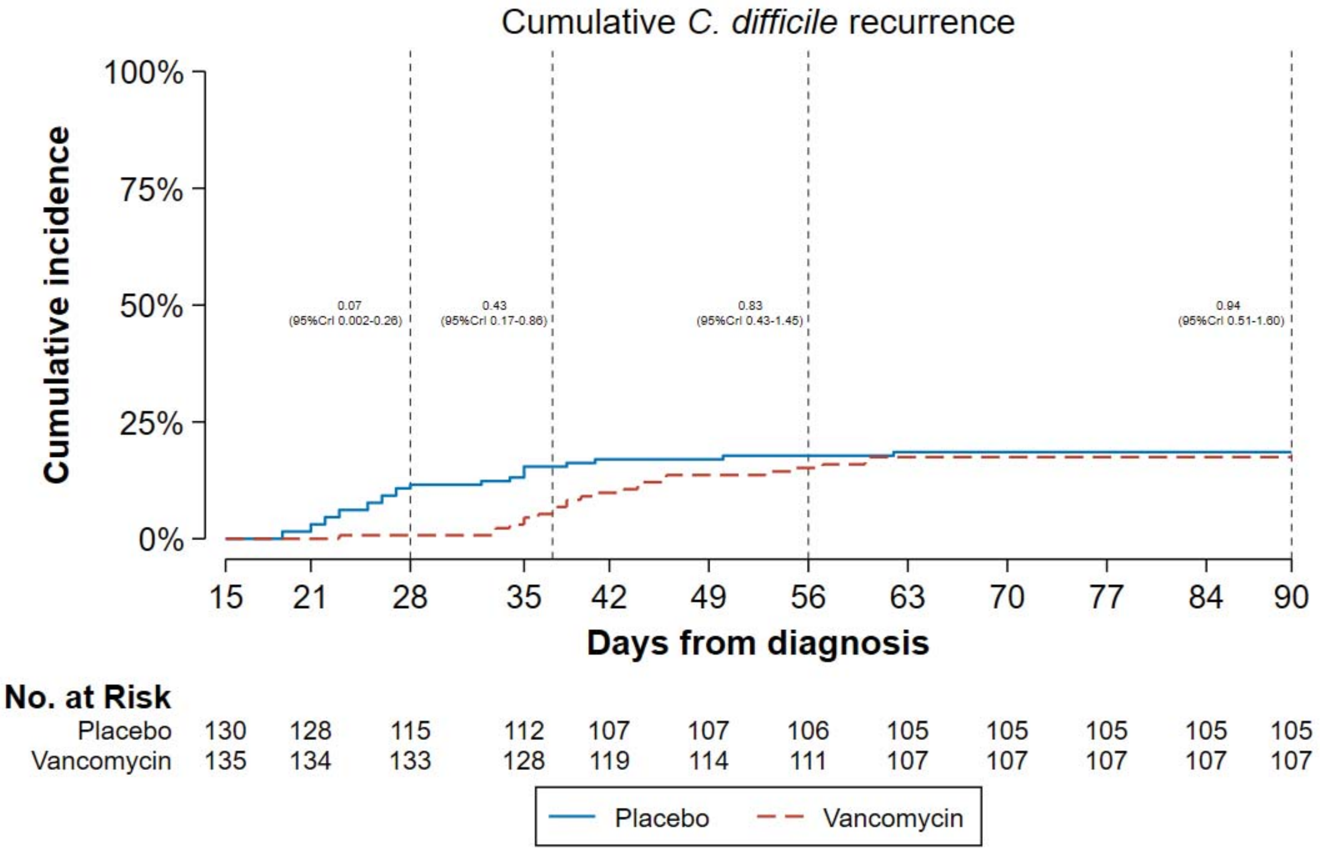

Supplementary Figure 2. Exploratory Subgroup Analysis

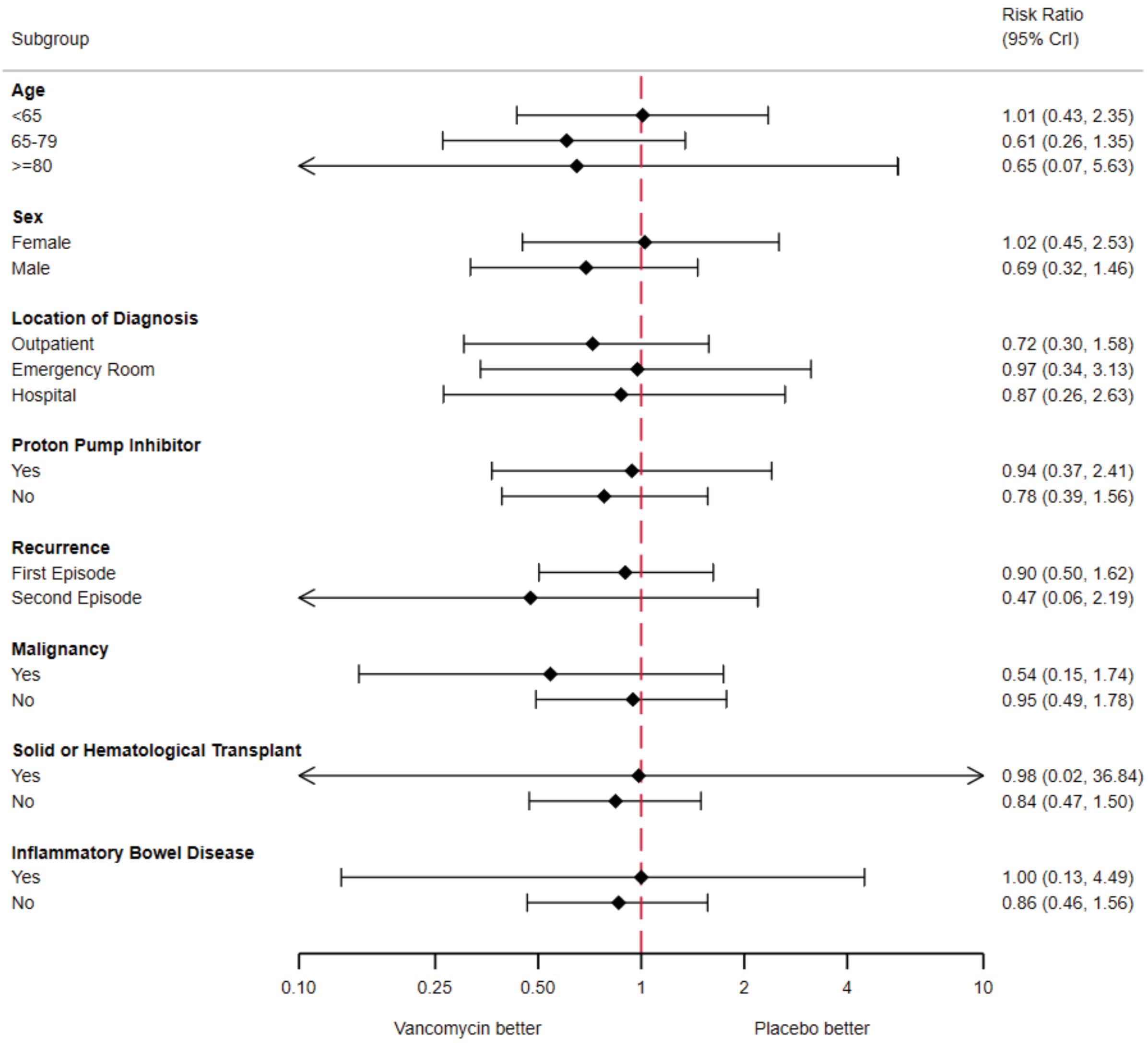

Supplement: Supplement 1. — Trial Protocol [file jamanetwopen-e2560495-s001.pdf]
