# Supplementary material for: Initial Vancomycin Taper for the Prevention of Recurrent Clostridioides difficile Infection: A Randomized Clinical Trial
Source: JAMA Netw Open. 2026 Feb 27;9(2):e2560495. doi: 10.1001/jamanetworkopen.2025.60495 (PMC12949445; doi:10.1001/jamanetworkopen.2025.60495)
Supplement: Supplement 2. — eTable 1. Restricted Mean Survival Time Analysis eTable 2. Per-Protocol Analyses and Recurrence Outcomes eFigure. Exploratory Subgroup Analysis eAppendix 1. Post Hoc Analysis: Antibiotic Re-exposure eAppendix 2. Post Hoc Analysis: Accounting for Site [file jamanetwopen-e2560495-s002.pdf]

Supplementary Online Content

McDonald EG, Butler-LaPorte G, Brophy JM, et al; TAPER-V Team. initial vancomycin taper for the prevention of recurrent *Clostridioides difficile* infection: a randomized clinical trial. *JAMA Netw Open.* 2026;9(2):e2560495. doi:10.1001/jamanetworkopen.2025.60495

eTable 1. Restricted Mean Survival Time Analysis

eTable 2. Per-Protocol Analyses and Recurrence Outcomes

eFigure. Exploratory Subgroup Analysis

eAppendix 1. Post Hoc Analysis: Antibiotic Re-exposure

eAppendix 2. Post Hoc Analysis: Accounting for Site

This supplementary material has been provided by the authors to give readers additional information about their work.

© 2026 McDonald EG et al. *JAMA Network Open*.

eTable 1. Restricted Mean Survival Time Analysis

Model Summary (difference of Restricted Mean Survival Time)			
	Coefficient	95% Confidence Interval	P-Value
Intercept	31.38	29.9-32.9	<0.001
Treatment	2.19	0.51-3.87	0.01
Stratification (First episode vs. First Relapse)	-1.44	-4.83 to 1.95	0.41
**Model Summary (ratio of Restricted Mean Survival Time)**			
	Exponentiated Coefficient	95% Confidence Interval	P-Value
Intercept	31.38	29.9-32.9	<0.001
Treatment	1.07	1.02-1.13	0.012
Stratification (First episode vs. First Relapse)	0.96	0.86-1.07	0.42

© 2026 McDonald EG et al. *JAMA Network Open*.

eTable 2. Per Protocol Analyses and Recurrence Outcomes

Characteristic	Vancomycin	Placebo	Adjusted Relative Risk (95% CrI)	Probability of Superiority
Per Protocol without vancomycin prophylaxis before day 28	(n=122)	(n=116)		
Recurrence of *C. difficile*				
Day 56 - Primary outcome	20 (16.4%)	22 (19.0%)	0.87 (0.49-1.52)	69.8%
Day 38 - Secondary outcome	9 (7.4%)	19 (16.4%)	0.45 (0.20-0.94)	98.4%
Day 90 - Secondary outcome	23 (18.9%)	23 (19.8%)	0.95 (0.56-1.64)	57.8%
Per Protocol without any vancomycin prophylaxis	(n=108)	(n=106)		
Recurrence of *C. difficile*				
Day 56 - Primary outcome	15 (13.9%)	18 (17.0%)	0.81 (0.42-1.53)	74.2%
Day 38 - Secondary outcome	7 (6.5%)	15 (14.2%)	0.44 (0.17-1.02)	97.2%
Day 90 - Secondary outcome	17 (15.7%)	19 (17.9%)	0.88 (0.47-1.59)	66.7%

© 2026 McDonald EG et al. *JAMA Network Open*.

eFigure. Exploratory Subgroup Analysis

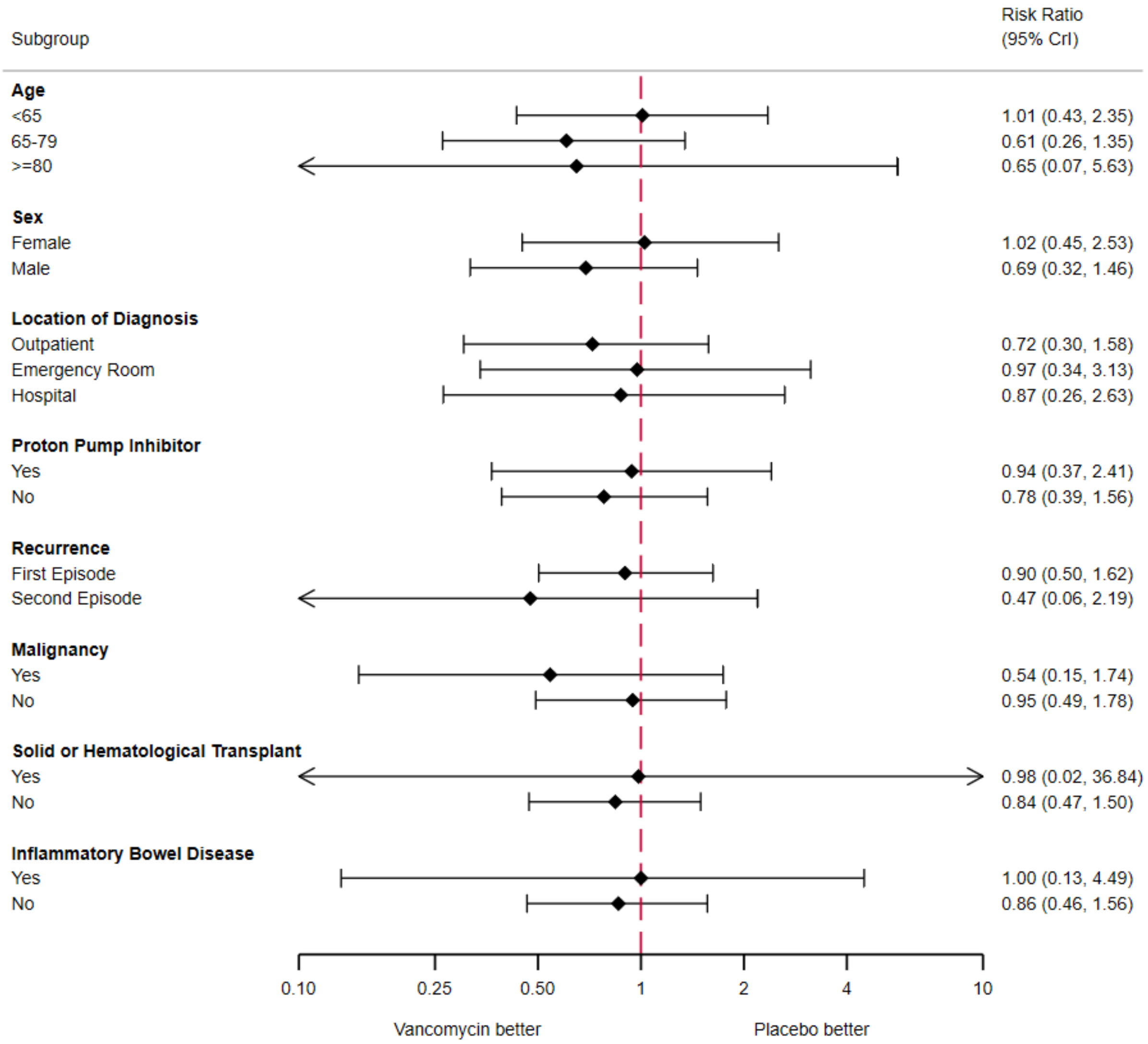

eAppendix 1. Post Hoc Analysis: Antibiotic Re-exposure

As requested by peer review, we conducted an analysis looking at the effect of downstream antibiotic exposure on the outcome. In patients who received systemic antibiotics, there were 2/54 recurrences (3.7%) versus 41/211 (19.4%). Adjusting for treatment status, stratum, and antibiotic re-exposure yielded the following results:

Treatment: RR 0.80 (95%CrI 0.44-1.33; probability of superiority 82.5%)
Antibiotic re-exposure: RR 0.19 (95% CrI 0.02-0.53)
Stratum: RR 1.16 (95% CrI 0.45-2.20)

© 2026 McDonald EG et al. *JAMA Network Open*.

eAppendix 2. Post Hoc Analysis: Accounting for Site

As requested by peer review, we conducted an analysis including site of enrollment in the generalized linear model.

Treatment: RR 0.83 (95%CrI 0.47-1.44; probability of superiority 74.1%)

© 2026 McDonald EG et al. *JAMA Network Open*.
