# Supplementary material for: Initial Vancomycin Taper for the Prevention of Recurrent Clostridioides difficile Infection: A Randomized Clinical Trial
Source: JAMA Netw Open. 2026 Feb 27;9(2):e2560495. doi: 10.1001/jamanetworkopen.2025.60495 (PMC12949445; doi:10.1001/jamanetworkopen.2025.60495)
Supplement: Supplement 3. — Nonauthor Collaborators [file jamanetwopen-e2560495-s003.pdf]

Supplemental Online Content: Nonauthor Collaborators
*First name, last name, and suffix (if applicable) are required and will appear in PubMed.

***Group Name(s): TAPER-V Team**

***First Name and Middle Initial(s)**	***Last Name**	***Suffix (eg, Jr, III)**	Academic Degrees	Institution	Location (city, state/province, country)	Role or Contribution, eg, chair, principal investigator	Group (if more than 1 Group listed in the byline) and/or Subgroup (eg, Steering Committee)
Claire J.	Lin			Vancouver General Hospital	Vancouver, British Columbi	Research Assistant	TAPER-V Team
Malaz J.	Idris		BSc	The Ottawa Hospital	Ottawa, Ontario, Canada	Research Assistant	TAPER-V Team
Adrienne K.	Chan		MD MPH	Sunnybrook Health Sciences Centre	Toronto, Ontario, Canada	Co-investigator	TAPER-V Team
Asgar	Rishu		MSc	Sunnybrook Health Sciences Centre	Toronto, Ontario, Canada	Coordinator	TAPER-V Team
Noelle R.	Yee		MSc	University Health Network	Toronto, Ontario, Canada	Research Assistant	TAPER-V Team
Maria	Kulikova		DPT	University Health Network	Toronto, Ontario, Canada	Research Assistant	TAPER-V Team
Jeff E.	Powis		MD MSc	Michael Garron Hospital	Toronto, Ontario, Canada	Co-investigator	TAPER-V Team
Maureen	Taylor		BSc	Michael Garron Hospital	Toronto, Ontario, Canada	Research Assistant	TAPER-V Team
Dominik	Mertz		MD MSc	McMaster University	Hamilton, Ontario, Canada	Chair	DSMB
Biyue	Dai		PhD	University of Minnesota	Minneapolis, Minnesota, U	member	DSMB
Zain	Chagla		MD	McMaster University	Hamilton, Ontario, Canada	member	DSMB
Jenine	Leal		PhD	University of Calgary	Calgary, Alberta, Canada	observer	DSMB
Elissa	Rennert May		MD MSc	University of Calgary	Calgary, Alberta, Canada	observer	DSMB
