# Supplementary material for: Initial Vancomycin Taper for the Prevention of Recurrent Clostridioides difficile Infection: A Randomized Clinical Trial
Source: JAMA Netw Open. 2026 Feb 27;9(2):e2560495. doi: 10.1001/jamanetworkopen.2025.60495 (PMC12949445; doi:10.1001/jamanetworkopen.2025.60495)
Supplement: Supplement 4. — Data Sharing Statement [file jamanetwopen-e2560495-s004.pdf]

Data Sharing Statement

McDonald. Initial Vancomycin Taper for the Prevention of Recurrent Clostridioides difficile Infection. *JAMA Netw Open*. Published February 27, 2026.
doi:10.1001/jamanetworkopen.2025.60495

Data
Additional Information: Trial Registration: ClinicalTrials.gov number NCT04138706
https://clinicaltrials.gov/study/NCT04138706
Data available: Yes
Data types: Deidentified participant data
How to access data: Data can be accessed upon reasonable request to the senior author Todd Lee at todd.lee@mcgill.ca
When available: beginning date: 01-01-2027, end date: 01-01-2028

Supporting Documents
Document types: None

Additional Information
Who can access the data: Non-industry academic researchers, clinicians, or policy makers
Types of analyses: To conduct secondary analyses or systematic reviews or to generate guidelines
Mechanisms of data availability: With investigator support within reason and an interinstitional data sharing agreement
